# Smart IoT with the hybrid evolutionary method and image processing for tumor detection

**DOI:** 10.1038/s41598-025-16042-0

**Published:** 2025-08-25

**Authors:** Yan Gao

**Affiliations:** School of Electrical and Mechanical Engineering, Xuchang University, Xuchang, 461000 Henan China

**Keywords:** Image processing, Tumor detection, Fog computing, IoT, Data placement, Health care, Health occupations, Medical research, Diseases

## Abstract

The primary objective of modern healthcare systems is to enhance public health by providing efficient, reliable, and well-structured solutions. Improving patient satisfaction through tailored medical services has driven rapid advancements in healthcare, leading to increased competition and system complexity. However, the expansion of healthcare services introduces challenges such as high data volume, latency, response time constraints, and security vulnerabilities. To address these issues, fog computing offers an effective solution by processing data closer to end devices, reducing latency, and enabling real-time responses. This research proposes a robust brain tumor detection framework within a fog-based smart healthcare infrastructure. The process begins with data placement leveraging an improved evolutionary technique for Image Processing (HETS-IP) to optimize fog node placement based on key parameters such as energy efficiency and latency. Specifically, the Particle Swarm Optimization (PSO) algorithm is enhanced with a direct binary encoding technique, in which solutions are represented as binary strings, making it suitable for problems where decisions are discrete. This approach allows efficient optimization in binary decision spaces and improves adaptability for complex placement problems. Once data placement is committed, the tumor detection framework is performed directly at fog nodes to enhance real-time processing. This phase will begin with preprocessing, where a bilateral filter is applied to reduce noise while preserving critical edge details. Next, feature extraction is utilized to derive statistical texture features, which capture diagnostic information essential for distinguishing between tumor types. The process continues by classification using a deep Convolutional Neural Network (CNN) with sequential architecture to classify tumors. Simulation results demonstrate that HETS-IP outperforms traditional evolutionary algorithms, including Ant Colony Optimization (ACO), Genetic Algorithm-Simulated Annealing (GASA), and Genetic Algorithm (GA). On average, HETS-IP reduces energy consumption by 5%, 9%, and 14% and decreases makespan by 4%, 6%, and 11%, respectively. Additionally, the proposed approach achieves an accuracy of 97% and a precision of 96%, ensuring highly reliable brain tumor detection.

## Introduction

The healthcare sector has grown quickly recently and has significantly increased both employment and revenue^1^. Only a few years ago, a hospital physical examination was required to diagnose illnesses and abnormalities in the human body. For the duration of their therapy, the majority of the patients had to remain in the hospital. In addition to raising healthcare costs, this put pressure on healthcare facilities in rural and isolated areas. The development of technology over the years has made it possible to use small devices, such as smartwatches, for health monitoring and the diagnosis of a variety of illnesses. Additionally, technology has changed the healthcare system from being hospital-centric to being patient-centric^2,3^. Many clinical studies, for instance, can be carried out at home without a healthcare provider’s assistance, including blood pressure, glucose, and so forth. Furthermore, sophisticated telecommunication technologies enable the transmission of clinical data from distant locations to healthcare facilities^4^. Healthcare facilities are now more accessible because of the utilization of these communication services and the quickly developing technology (such as fog computing, wireless sensing, IoT, big data analysis, machine learning, and mobile computing).

**Fig. 1:**
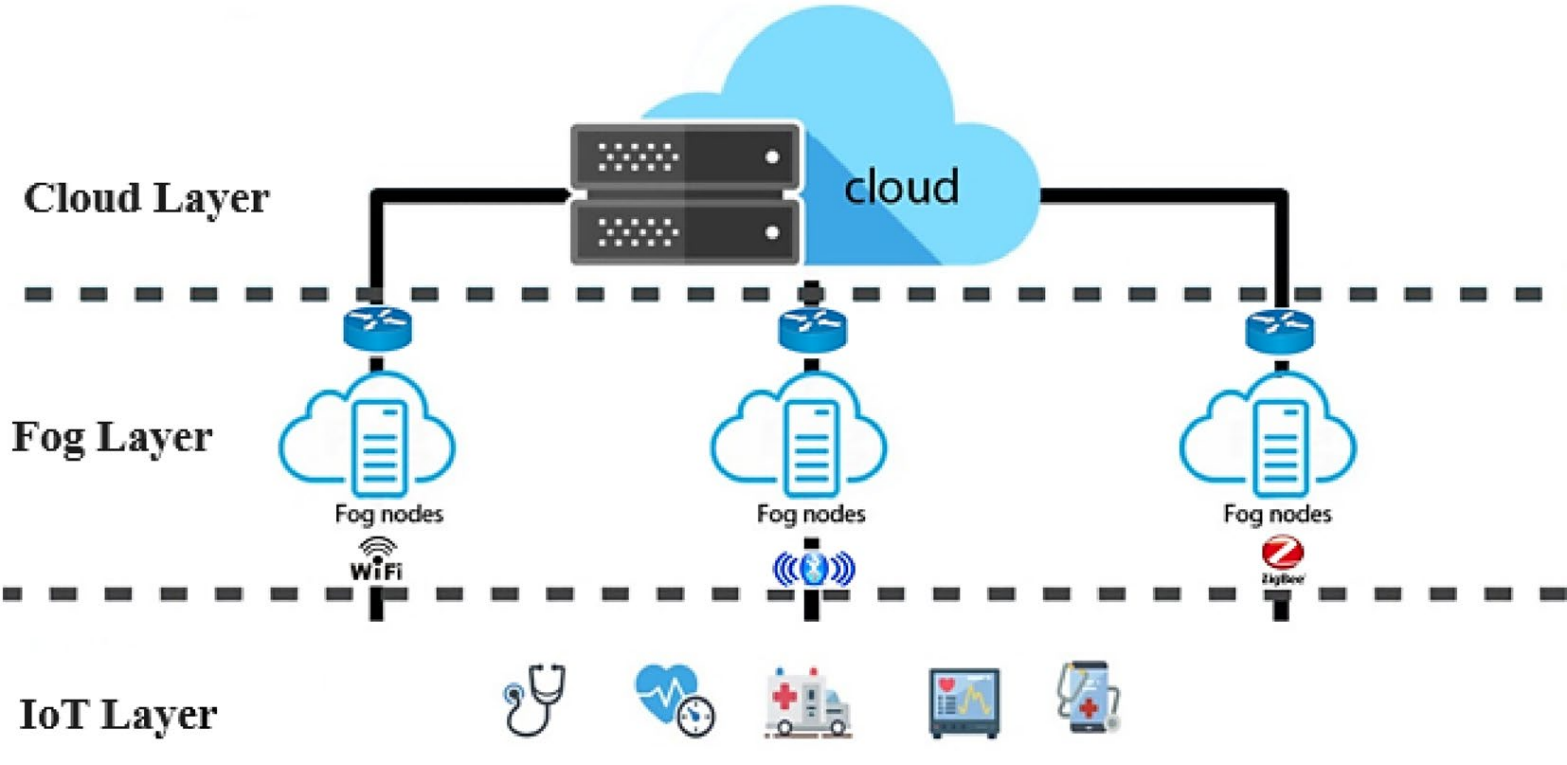
The structure of a healthcare system with using fog and cloud computing.

The fog computing paradigm, an extension of cloud computing, plays a crucial role in the functioning of IoT applications by addressing key healthcare challenges such as latency in data processing, limited bandwidth in remote areas, and the need for real-time decision-making. Specifically, fog computing decentralizes computation and storage to the network edge, closer to data sources like IoT devices, thereby reducing delays and alleviating pressure on centralized fog-cloud systems^5^. This directly impacts healthcare delivery by enabling faster diagnostics (e.g., real-time monitoring of vital signs), improving accessibility for patients in underserved regions, and supporting cost-effective, patient-centric care through efficient data handling^6,7^. In order to establish a connection between IoT devices and the cloud layer, the solution architect intends to deploy fog nodes at the network edge, as seen in Fig. 1 of the hierarchical model. These fog nodes possess distinct computational and storage capabilities.

The Internet of Things has not only increased human independence but also expanded human interaction with the outside world. With the aid of cutting-edge algorithms and protocols, IoT has emerged as a significant force in international communication. Numerous gadgets, wireless sensors, household appliances, and technological devices are connected to the Internet using it^4^. IoT applications are seen in the following domains: healthcare^1,8^, homes^9^, cars, and agriculture^5^. The Internet of Things is becoming more and more popular because of its advantages, which include greater future event prediction, reduced costs, and improved accuracy. The quick IoT revolution has also been aided by greater understanding of software and applications, the advancement of computer and mobile technologies, the accessibility of wireless technology, and the growing digital economy^10^. Using various communication protocols like Bluetooth, Zigbee, IEEE 802.11 (Wi-Fi), and others, the Internet of Things devices have been combined with other physical devices to monitor and share data. Sensors, whether wearable or installed on the human body, are utilized in healthcare applications to gather physiological data from patients’ bodies, including temperature, pressure, electrocardiogram (ECG), electroencephalogram (EEG), and more^11^. It is also possible to capture environmental data like temperature, humidity, time, and date. This data aids in drawing accurate and significant conclusions about the patients’ medical situations. Since a lot of data is collected and recorded from a range of sources, data storage and accessibility are also crucial in IoT systems. Physicians, caregivers, and other authorized individuals have access to the data from the aforementioned sensing devices. Sharing this data with healthcare providers via a server or cloud enables prompt patient diagnosis and, if required, medical action. The communication module, patients, and users all work together to provide safe and efficient transmission. The majority of IoT systems execute user control, data visualization, and apprehension through a user interface that serves as a dashboard for medical providers. The literature that has documented the advancements of IoT systems in healthcare monitoring, control, security, and privacy has found a wealth of research^12^. These achievements demonstrate the IoT’s efficacy and promising future in the healthcare industry. However, preserving the quality-of-service matrices, which include availability, pricing, security, privacy of information sharing, and reliability, are the primary considerations while creating an IoT device.

Even though smart healthcare and IoT systems are growing quickly, current data placement tactics in fog computing settings still have problems with optimizing latency, saving energy, and finding tumors in real time. Many methods only look at resource allocation or data placement, but not at the same time to improve the performance of the whole system. Also, classical evolutionary algorithms frequently have trouble converging quickly in healthcare IoT networks, which are usually high-dimensional and discrete problem spaces. In medical situations where time is of the essence, like finding brain tumors, these delays are intolerable^13^. This study was inspired by the urgent need for a framework for detecting tumors that is quick, energy-efficient, and accurate in a fog-based smart healthcare setting. The suggested HETS-IP technique fills this gap by using a modified Particle Swarm Optimization (PSO) algorithm along with direct binary encoding to optimize the placement of data and the allocation of virtual machines at the same time. This dual optimization enables more effective handling of the exponentially generated IoT data and their resource constraints within the fog infrastructure, addressing the unique complexities of IoT-fog systems. Distributing the data from IoT nodes to the fog infrastructure is essential, considering the unique computation and communication attributes. We are compelled to tackle these challenges by the application of an evolutionary approach. Algorithms based on PSO require fewer parameters for calibration. By considering the interaction of particles, it can achieve the ideal solution. However, when faced with a multidimensional search space, PSO typically converges on the global optimum. This way, it makes sure that latency is lower, energy use is better, and real-time diagnosis is possible, which is a big gap in current IoT-fog medical frameworks.

The subsequent sections of the paper are structured as follows: The “Related work” section examines the current research on data placement and IoT-based healthcare systems. The “Problem formulation” section delineates the system model and the formulation of the problem. The suggested methodology is detailed in the “Proposed algorithm” section. The “Implementation” section delineates the findings of experimentation and comparison. The “Conclusions” section summarizes the paper and delineates future research directions.

## Related works

The medical field has evolved into a highly competitive domain. Patient satisfaction can be improved by considering patient demands during service design and delivery, particularly by reviewing patients’ medical histories^14^. Moreover, accessing medical facilities has become challenging due to the prevalence of infectious and epidemic diseases like COVID-19, along with high costs, long distances, and quarantine factors that disproportionately affect elderly and disabled individuals^15,16^. In this regard, the architecture of the smart hospital system (SHS) enables rapid access to patient data and expedites the treatment process^17^. This design facilitates automatic tracking and monitoring of hospital practitioners, biomedical equipment, and patients^18^. It also continuously records variations in patients’ vital physiological signs and environmental conditions. Using a configurable web service based on representational state transfer, the collected parameters are sent to a control center, allowing both local and remote access^19,20^. The proposed SHS can be validated through two primary scenarios: one involving patient monitoring and the other addressing emergency management in the event of real-time patient falls^21^. The use of healthcare information technology (HIT) in hospitals and related institutions is deeply influenced by safety concerns and healthcare quality, which encompasses medical care, general care, ambulatory services, and specialized disease care. Thousands of sensors may be installed in hospitals and smart buildings to record operational metrics such as patient condition, blood pressure, ECG, blood glucose levels, humidity, and temperature. These sensors periodically transmit data to local storage servers, and after processing, relevant insights about a patient’s condition are sent to physicians or caregivers^22^. In emergencies such as heart attacks or sudden spikes in blood pressure, real-time data analysis and communication are critical, and cloud architectures are often insufficient to meet these time-sensitive needs^1^. In such cases, fog computing bridges the gap between the sensing layer and healthcare centers. Fog nodes provide a scalable, mobile, and reliable computational and storage infrastructure that supports various devices, including desktop PCs, smartphones, and tablets^23^.

A study in^24^ proposed a comprehensive multi-layer architecture for IoT-enabled e-health systems integrating cloud computing, fog computing, and edge devices. This architecture is designed to address challenges related to latency, data heterogeneity, and real-time processing. The study also identified issues including scalability, data management, interoperability, privacy, and security, and emphasized the need for networks with high bandwidth, low latency, and user-friendly interfaces. However, recent publications were not reviewed, and the methodology for selecting sources was not transparent. Another study^25^ emphasized the role of nanocomposites in healthcare sectors, such as food processing, biomedical applications, and nano-carrier technologies. The study focused on polymer nanocomposites used in diagnostics and treatment, along with their various processing techniques. However, the selection criteria for reviewed papers lacked clarity, and the publication dates were not disclosed. The investigation in^26^ analyzed industry sources, including websites, white papers, and podcasts, to provide a structured overview of fog computing in healthcare. It highlighted technological standards, current challenges, and system functionalities such as health monitoring and specialized care for defensive applications. In^27^, a comprehensive survey was conducted on the role of IoT and cloud computing in healthcare systems. The concept of Cloud IoT-Health was introduced, integrating these technologies for applications such as medication tracking, telemedicine, and smart hospitals. An evaluation of open-source projects was included. While the study provided useful insights, it did not cover fog computing, and the paper selection process was not clearly explained. A separate review^28^ discussed the Cloud of Things (CoT) in healthcare, examining its architecture and platforms. The findings showed that most studies neglected energy efficiency considerations, especially in scenarios requiring the balancing of delay, quality of service, and energy consumption. This study, too, excluded fog-related research, lacked recent literature, and was limited in scope. Additional surveys, such as^29^, examined cybersecurity concerns to propose a comprehensive security model for electronic health records. Another study^30^ reviewed recent research trends in assisted healthcare environments, particularly those supporting cardiovascular disease monitoring and diagnostics. A review in^31^ focused on IoT-based cardiovascular health systems, identifying critical challenges that must be overcome to innovate in heart care technologies. In^32^, data management strategies for healthcare systems using IoT and cloud infrastructures were analyzed. A review in^33^ addressed data privacy challenges in constrained healthcare settings. The study presented a deep analysis of privacy-preserving mechanisms and concluded that a balanced approach is required to protect patient data. However, it did not discuss fog computing. Finally, a study in^34^ categorized fog computing applications in healthcare, outlined relevant use cases, and described the associated devices and networks. While it demonstrated the potential of fog computing, it lacked comparative analysis and did not identify unresolved issues or future research directions. In^35^, a model called UCFNNet is proposed in order to improve the accuracy of ulcerative colitis assessment. The system includes an extensive lesion learner, which is focused on identifying specific patterns of inflammation in endoscopic images, and a noise suppression gating process, which reduces irrelevant noise. These components work together to improve the segmentation and evaluation of inflamed regions in the colon. The tumor identification and visualization of the small arteries requires rapid and precise imaging. In^13^, a method based on a deep learning algorithm is proposed to speed up the denoising process. The traditional methods may deliver noisy images and require long processing times. The new model is efficient in image improvement without losing important information, which makes the technology more relevant in real-time medical applications. Detection of hypoxic cancer cells is important in cancer studies. In^36^, the authors describe a highly efficient fluorescent probe that uses a nitroaryl group to bind to nitro reductase. On contact with nitro reductase, the probe fluoresces, which enables the use of imaging techniques to assess hypoxic cells in tumors. This could help to understand how tumors act and create better diagnostic tools.

Authors in^37^ presented a thorough review of how to find Down syndrome using new technology, concentrating on how fetal screening methods and smart diagnostic models have improved. Their work shows how important it is to find problems early and how image processing and machine learning may help make diagnoses better. Also, another work in^38^ has suggested EURI, a deep ensemble design for segmenting and detecting oral lesions. This shows how successful ensemble tactics can be in making difficult medical imaging jobs easier to find and classify. Even though these studies are about different areas of medicine, they all show how important it is to have strong, real-time detection frameworks for making clinical decisions. They also highlight the need to build high-performance models like the HETS-IP that was suggested. A work in^39^ proposed a modified convolutional neural network for medical image classification within a fog-cloud architecture. The model was evaluated on pandemic-related X-ray datasets and achieved nearly 99.9 percent accuracy. The study emphasized reducing model complexity to fit the limitations of edge devices. It showed that lighter CNNs could match or exceed the performance of more complex models like VGG16. These results support the choice of CNNs for real-time medical processing in fog environments. Authors in^40^ presented a federated learning-based system named FedHealthFog for predicting heart disease in fog networks. The model allows collaborative learning across medical sites while protecting patient privacy. Although promising, it introduces high communication overhead and synchronization challenges that can affect response time. These limitations highlight the advantages of HETS-IP, which avoids such delays by operating locally on fog nodes. This makes it better suited for emergency diagnosis and continuous monitoring. another work in^41^ reviewed the current state of federated learning in healthcare and identified significant implementation challenges. It discussed problems such as data imbalance across clients, hardware variability, and the high cost of secure communications. The review concluded that while federated learning is valuable, it remains difficult to scale efficiently in clinical settings. In contrast, HETS-IP reduces complexity by processing data locally, which lowers delay and system dependence on network stability. For predicting cardiovascular events in fog environments, the authors in^42^ proposed a hybrid architecture combining CNN, BiLSTM, and reinforcement learning. The system used digital twins to simulate and adapt to device conditions in real time. It achieved strong accuracy and operated well under fluctuating load conditions. Authors in^43^ introduced a transformer-based model applied within a federated learning framework for handling multi-modal healthcare data. It addressed client diversity and cross-device inconsistencies that challenge global training. The approach improves generalization but requires large computational resources not typically available on fog nodes.

While the reviewed literature has contributed significantly to the development of IoT and fog-based healthcare systems, several critical problems remain unresolved:

- *Isolated Optimization* Most studies conducted so far have focused on resource allocation or data placement in isolation, without considering how they interact with each other. In real-time healthcare scenarios, this segmentation results in a less efficient and less responsive system overall.
- *Delay Constraints* A lot of traditional models struggle to scale when applied to large, mixed datasets generated in modern healthcare settings. Inefficient scheduling and poor resource utilization exacerbate latency-sensitive apps, such as brain tumor detection, which are particularly affected.
- *Not Handling Discrete Search Spaces Well Enough* Traditional evolutionary algorithms like GA and ACO often fail to perform optimally on high-dimensional, discrete decision issues like allocating data tasks in foggy settings. They tend to converge prematurely and lack the adaptability required in changing IoT settings.
- *Not Being Able to Work in Real Time* Cloud-based solutions are robust, but they generally cannot provide the low latency necessary for important diagnostic operations. These systems typically slow down data transmission and processing, making them unsuitable for emergency medical care.
- *Limited use of Learning-Based Classification* Some studies employ machine learning or deep learning to detect tumors, but few combine these classifiers with the best fog node placement procedures. As a result, diagnosis workflows can’t be accelerated from start to finish.

To overcome these challenges, we developed the HETS-IP system, which integrates evolutionary optimization for data placement with deep learning-based tumor categorization directly at fog nodes. A survey of the existing works in fog/IoT-based healthcare systems is shown in Table 1.

**Table 1:** Comparative survey of related works in IoT and Fog-based healthcare systems.

References	Method	Application	Strengths	Limitations
^24^	Multi-layer fog-cloud-edge architecture	IoT-enabled e-health	Real-time processing, layered architecture	No recent literature; lacks source selection transparency
^25^	Polymer nanocomposites	Diagnostics, treatment	Material-level innovation	Unclear paper selection criteria
^26^	Review of fog computing standards	Health monitoring systems	Focus on tech standards and functionalities	No experimental validation
^27^	Cloud-IoT integration	Telemedicine, smart hospitals	Covers multiple applications	Excludes fog computing
^28^	Cloud of Things (CoT)	Healthcare platforms	Reviews energy constraints	Ignores fog-layer tradeoffs
^29^	Cybersecurity model	EHR systems	Proposes robust security measures	Lacks fog computing focus
^30^	IoT-based cardiovascular monitoring	Assisted health environments	Highlights diagnostic use cases	Lacks generalizability across diseases
^31^	Cardiovascular health systems	Heart disease analytics	Identifies critical technical challenges	No focus on fog or energy-aware systems
^34^	Use-case-based fog computing review	Healthcare use cases	Good overview of fog applications	No gap identification, lacks performance comparison
^35^	Deep learning (UCFNNet)	Ulcerative colitis assessment	Effective feature extraction and segmentation	Not integrated with fog-layer data handling
^13^	Deep learning denoising	Real-time medical imaging	Preserves image fidelity in MRI/ultrasound	Not optimized for time-critical fog computing
^36^	Hypoxia probe for tumor imaging	Fluorescence-based cancer imaging	Enhances diagnostic imaging	Requires specific biological conditions, not scalable in fog-IoT environments
^37^	Survey of Down syndrome detection technologies	Prenatal diagnostics	Reviews modern imaging and AI tools	Focused on a single diagnostic field
^38^	Deep ensemble model (EURI)	Oral lesion segmentation	Strong segmentation performance	Limited to oral image classification
^39^	Modified CNN in fog-cloud framework	Pandemic X-ray imaging	High-accuracy, fog-ready CNN model	Focuses on single imaging context
^40^	Federated learning on fog nodes (FedHealthFog)	Heart disease prediction	Privacy-preserving, real-world deployment	Communication and synchronization overhead
^41^	Critical review of federated learning in healthcare	Healthcare AI systems	Identifies practical barriers, future directions	Limited feasibility in resource-constrained deployments
^42^	Hybrid CNN-BiLSTM with digital twins	Cardiovascular prediction	High accuracy and dynamic fog adaptation	Complex system integration required
^43^	Transformer with federated learning	Multi-modal data fusion	Strong generalization, cross-device learning	High resource demands, not fog-compatible

## Problem formulation

**Fig. 2:**
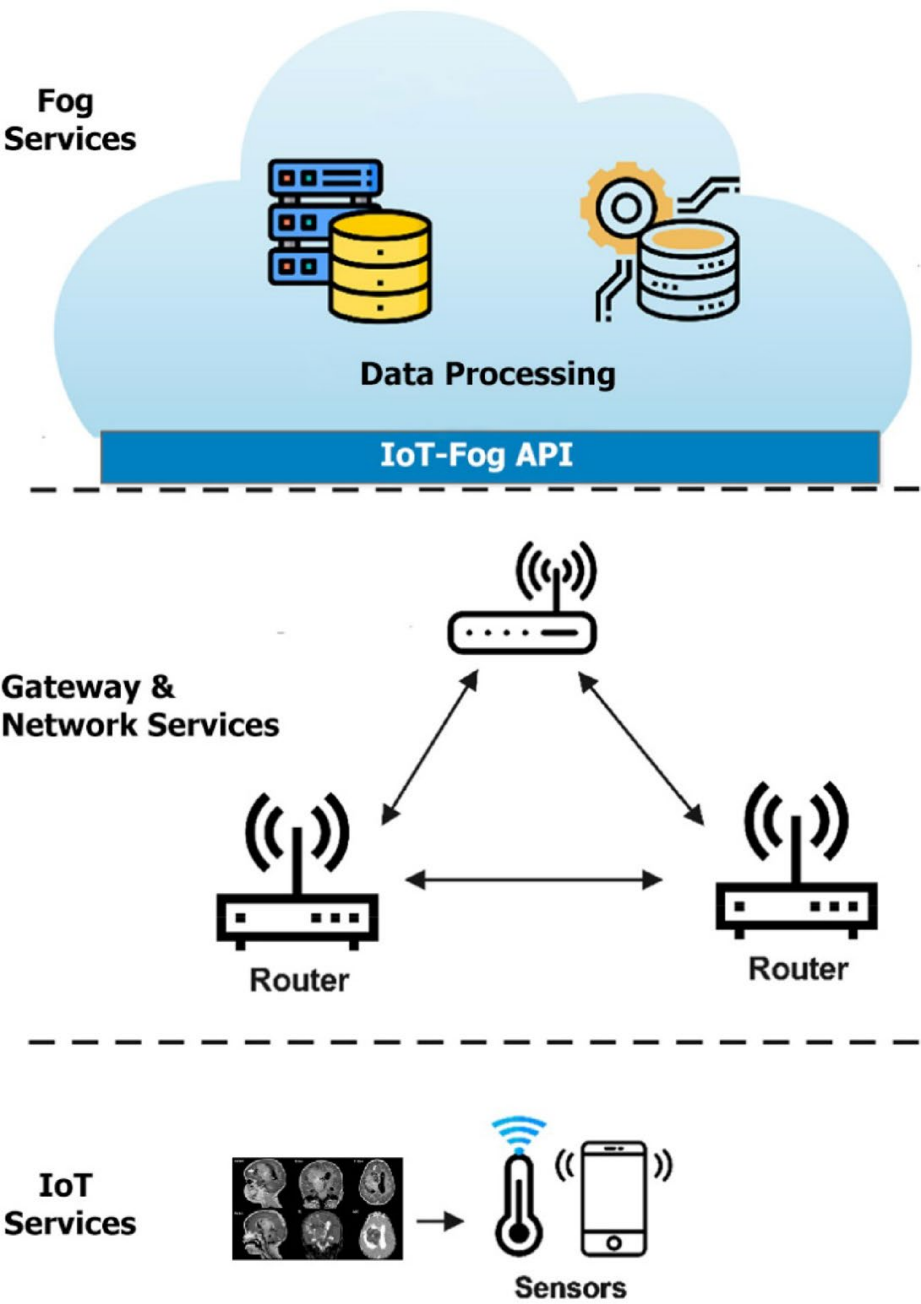
The structure of proposed method.

The diverse data distribution throughout a healthcare system’s computing environment is depicted in Fig. 2. The system consists of a set of IoT terminal devices T = {t_1_, t_2_, t_3_, …, t_i_, …, t_n_}, where each device t_i_ generates data tasks A_i_. These tasks are placed on a set of fog nodes FN = {FN_1_, FN_2_,…,FN_m_}, each characterized by computational resources R_j_, storage capacity C_j_, and network bandwidth Nj. The placement decision is denoted by a binary variable r_ij_(t) ∈ {0,1}, where r_ij_(t) indicates that task Ai is placed to fog node FN_j_ at time slot t.

Each task A is defined by attributes P_A_ = {S_i_, β_i_, M_i,max_, M_i,exp_, Pi} where.

- S_i_ is the size of the task data
- β is the computational intensity (CPU cycles per bit)
- M_i,max_ is the maximum tolerable latency
- M_i,exp_ is the expected completion time
- P_i_ is the priority weight of the task

Each terminal device t_i_ has attributes Q_t_ = {p_tr_, p_te_, b_i_, w_td_, w_ed_, E_r_}, where p_tr_ indicates the transmission power of t_i_, p_te_ represents the idle power of t_i_, and b_i_ is a binary variable indicating the mobility status of t_i_. The parameters w_td_ and w_ed_ signify the delayed weight and energy consumption weight of t_i_, respectively. If b_i_ = 1, it denotes that t_i_ is a mobile device with limited energy. Considering the energy consumption aspect during data placement, we set w_td_ = 0.8 and w_ed_ = 0.2. In contrast, if b_i_ = 0, it signifies that t_i_ is a static device with infinite energy, leading to the configuration of w_td_ = 1 and w_ed_ = 0. E_r_ denotes the remaining energy of t_i_.

### Latency model

The total latency for processing task Ai on fog node FN_j_ at time slot t, denoted L_ij_(t), comprises three components: transmission latency (L_tr,ij_(t)), queuing latency (L_q,ij_(t)), and computation latency (L_comp,ij_(t)). The model accounts for dynamic network conditions and task prioritization. The transmission latency is calculated as Eq. (1).

1 $${L}_{tr, ij}= \frac{{S}_{i}}{{K}_{ij}\left(t\right)}$$

where k_ij_(t) is the data transmission rate between device t_i_ and fog node FN_j_, modeled using the Shannon-Hartley theorem in Eq. (2).

2 $${k}_{ij}\left(t\right) = {N}_{j}\left(t\right) {\text{log}}_{2} \left(1 + \frac{{p}_{tr,i}\left(t\right) \cdot {g}_{ij}\left(t\right)}{\epsilon \left(t\right)}\right)$$

where g_ij_(t) is the channel gain, ϵ(t) is the noise power, and N_j_(t) is the available bandwidth. The queuing latency accounts for the waiting time due to other tasks at fog node FN_j_ is as Eq. (3).

3 $${L}_{q,ij}\left(t\right)={\sum }_{k\ne i,{r}_{kj}\left(t\right)=1}\frac{{S}_{k}\cdot {\beta }_{k}}{{R}_{j}\left(t\right)}\cdot \frac{1}{{P}_{k}}$$

where P_k_ is the priority weight of task A_k_, ensuring that high-priority tasks (e.g., critical health-care data) experience reduced queuing delays. The computation latency is given by Eq. (4).

4 $${L}_{comp,ij}\left(t\right)=\frac{{S}_{i}\cdot {\beta }_{i}}{{R}_{j}\left(t\right)}$$

where R_j_(t) is the computational resource of fog node FN_j_.

Therefore, the total latency is calculated as Eq. (5).

5 $${L}_{ij}\left(t\right)={L}_{tr,ij}\left(t\right)+{L}_{q,ij}\left(t\right)+{L}_{comp,ij}\left(t\right)$$

To ensure real-time processing, the model enforces a constraint as Eq. (6).

6 $${L}_{ij}\left(t\right)\le {M}_{i,{\text{max}}},\hspace{1em}\forall i,j\text{ where }{r}_{ij}\left(t\right)=1$$

### Energy consumption model

The total energy consumed by terminal device ti is categorized into the transmission energy E_tr,i_(t), idle energy Eidle,i(t), and placement energy E_off,i_(t). The model is designed such that it adapts the power control mechanism and takes into consideration the energy used to placement tasks to fog nodes.

The transmission of energy is calculated as Eq. (7).

7 $${E}_{tr,i}\left(t\right)={p}_{tr,i}\left(t\right)\cdot {L}_{tr,ij}\left(t\right),\hspace{1em}\text{if }{r}_{ij}\left(t\right)=1$$

The idle energy, incurred when the device is not transmitting, is calculated as Eq. (8).

8 $${E}_{idle,i}\left(t\right)={p}_{idle,i}\left(t\right)\cdot \left({T}_{\text{slot}}-{L}_{tr,ij}\left(t\right)\right)$$

where T_slot_ is the duration of the time slot.

The placement energy, which accounts for the energy consumed by the fog node to process the task, is calculated by Eq. (9).

9 $${E}_{off,i}\left(t\right)={\upkappa }_{j}\cdot {S}_{i}\cdot {\upbeta }_{i}\cdot {f}_{j}^{2}$$

where κ_j_ is the energy coefficient of fog node FN_j_, and f_j_ is the processing frequency of the fog node.

The total energy consumption for device t_i_ is calculated by Eq. (10).

10 $${E}_{i}\left(t\right)={\sum }_{j}{r}_{ij}\left(t\right)\cdot \left({E}_{tr,i}\left(t\right)+{E}_{off,i}\left(t\right)\right)+{E}_{idle,i}\left(t\right).$$

An energy constraint ${E}_{i}\left(t\right)\le {E}_{r,i},\forall i,$ ensures that the device does not deplete its battery.

### Objective function

The objective is to place and assign several data to dedicated fog nodes by employing a specific placement methodology. The objective is to reduce the duration of data completion while minimizing the energy consumption of terminal devices in meeting their data demands. In order to enhance efficiency and effectively address the matter at hand, the following assumptions are posited:

- There exists a lack of interdependence across data, with each data functioning autonomously from the others.
- Data are not multi-assignable, and each data can only be allocated to a single fog node.
- Furthermore, the calculation approach does not account for the mobility of terminal equipment.
- Each fog node remains stationary, and once a data commences execution, it does not undergo any interruptions.

The objective is to minimize a weighted combination of total latency and energy consumption across.

all tasks and devices, as formulated as Eq. (11).

11 $${\text{minimize}}\hspace{1em}F={\sum }_{i}{\sum }_{j}{r}_{ij}\left(t\right)\cdot \left({w}_{td,i}\cdot {L}_{ij}\left(t\right)+{w}_{ed,i}\cdot {E}_{i}\left(t\right)\right)$$

subject to:

${\sum }_{j}{r}_{ij}\left(t\right)\le 1,\hspace{1em}\forall i$ Each task is assigned to at most one fog node.

$${r}_{ij}\left(t\right)\in \{\text{0,1}\},\hspace{1em}\forall i,j$$ Binary decision.

${L}_{ij}\left(t\right)\le {M}_{i,{\text{max}}},\hspace{1em}\forall i,j$ where r_ij_(t) = 1: Latency constraint.

$${E}_{i}\left(t\right)\le {E}_{r,i},\hspace{1em}\forall i$$ Energy constraint.

${\sum }_{i}{r}_{ij}\left(t\right)\cdot {S}_{i}\cdot {\beta }_{i}\le {R}_{j}\left(t\right),\hspace{1em}\forall j$ Fog node resource constraint.

## Proposed algorithm

**Fig. 3:**
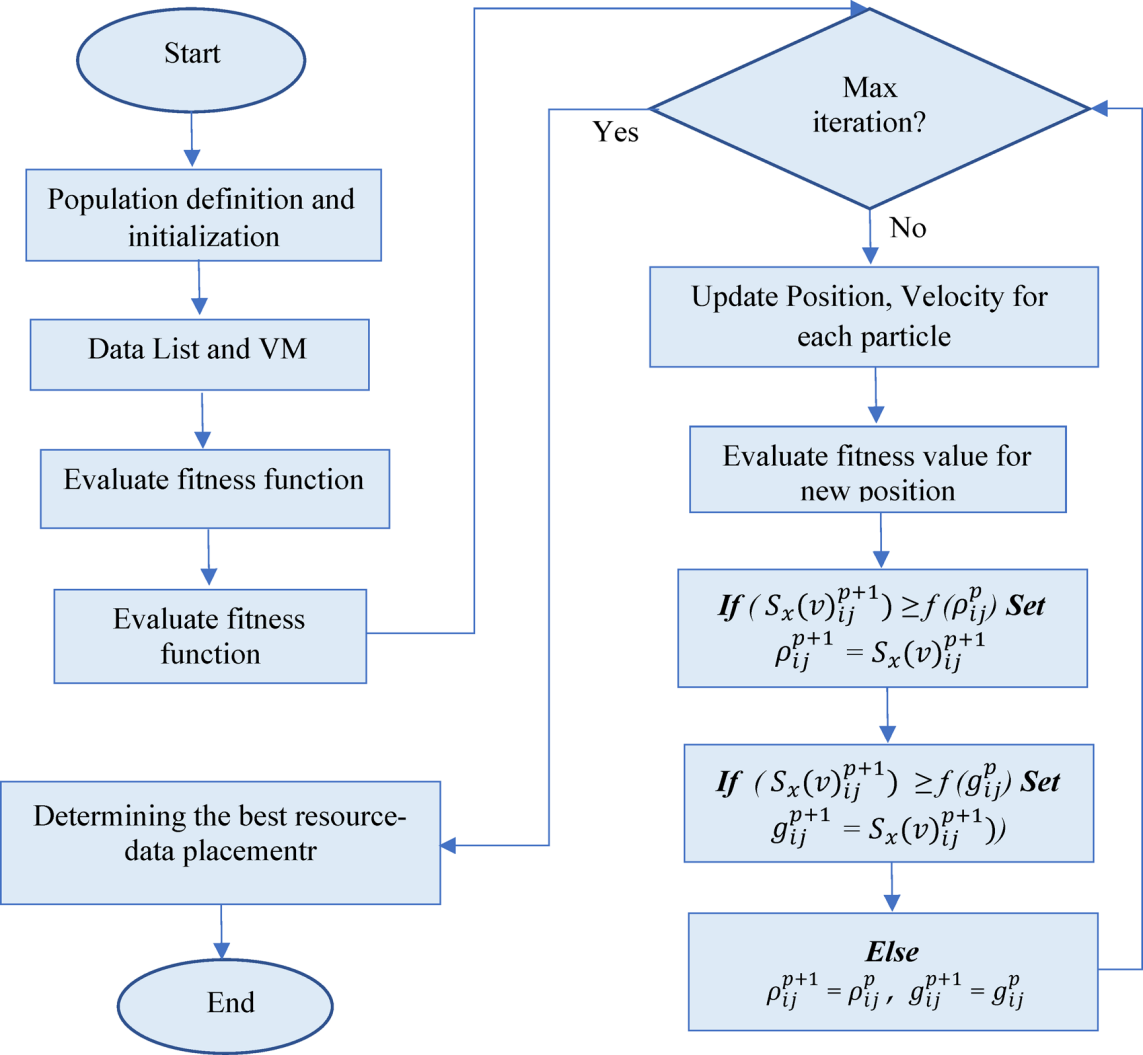
The flowchart of MPSO algorithm.

Data placement constitutes a complicated, multidimensional, nonlinear combinatorial optimization challenge with multiple objectives. The incorporation of several variables and constraints into the objective function complicates the identification of the optimal solution by polynomial methods. To address these issues, we employ the modified PSO (MPSO) algorithm. Our solution, grounded in the PSO approach, optimally positions data within their appropriate nodes. The PSO algorithm offers an efficient and rapid solution for intricate optimization problems; nevertheless, its usefulness is constrained to the distinct features of the fog computing task placement problem^44^. The intrinsic granularity of the parameters necessitates the application of discretization techniques in the standard PSO algorithm. To overcome this limitation and redefine the parameters of particle positions and velocities, an enhanced discrete particle MPSO algorithm is employed to address the data placement issue efficiently. Figure 3 illustrates the flowchart of the MPSO algorithm.

### Initialization

Our study utilizes a direct binary encoding method, a representation strategy in which the particle’s position is defined as a binary string composed of 0s and 1s. Each bit in this string corresponds to a specific decision variable. For example, whether a given data task is assigned (1) or not assigned (0) to a particular fog node. This technique is especially well-suited for discrete optimization problems like task placement, where solutions must satisfy binary constraints^45^. The velocity values, although continuous in [0,1], are interpreted as probabilities and mapped to binary positions via a sigmoid transformation. This mapping ensures that the PSO algorithm remains effective even in non-continuous solution spaces. The positional representation in binary encoding is illustrated by.

${x}_{i}= \{{x}_{\left\{i1\right\}}, {x}_{\left\{i2\right\}}, \dots , {x}_{\left\{id\right\}}\}, {x}_{\left\{ij\right\}}\in \{0, 1\}$ where x_i_​ represents the binary position of the particle i in the solution space, and d is the dimension of the data placement problem (number of data).

### Velocity and position updates

In standard PSO, positions are updated based on velocity values. For binary PSO, velocities are treated as probabilities, and the position update follows a sigmoid transformation to probabilistically flip the position values from 0 to 1 (or vice versa)^40^. The velocity update is shown in Eq. (12).

12 $${v}_{\left\{ij\right\}\left(t+1\right)}= w \cdot {v}_{ij}\left(t\right)+ {c}_{1}\cdot {r}_{1}\cdot \left({p}_{ij}^{best}- {x}_{ij}\left(t\right)\right)+ {c}_{2}\cdot {r}_{2}\cdot \left({g}_{j}^{best}- {x}_{ij}\left(t\right)\right)$$

Here, v_ij_(t) is the velocity of particle i at iteration t for data j, w is the inertia weight, c1and c2​ are acceleration coefficients, and r1​, r2​ are random values between 0 and 1. ${p}_{\left\{ij\right\}}^{best}$ and ${g}_{j}^{best}$ represent the personal and global best positions, respectively. Also Eq. (13) demonstrate the utilization of the sigmoid function S to represent the position vector of the particles, where the r_ij_ values are treated as binary variables^46^.

13 $$s\left({v}_{ij}\right)= \frac{1}{1+ {e}^{{v}_{ij}}}$$

The particle will update using Eq. (14).

14 $${r}_{ij}^{p+1}= \left\{\begin{array}{c}1, {r}_{3}< {{S}_{x}(\nu }_{ij}^{p+1})\\ 0, {r}_{3} \ge {{S}_{x}(\nu }_{ij}^{p+1})\end{array}\right.$$

where k is the number of iterations; pij and gij are the optimal and ideal positions of the j-th and j-th particles, respectively; c1 and c2 are acceleration coefficients; ν*ij* is the particle’s current velocity in the k-th iteration; and r1, r2, and r3 are random variables in the interval [0, 1].

### Contraction factor (η)

The inertia parameter, denoted as η, plays a crucial role in shaping the exploration of particles within both global and local search regions. By increasing the contraction factor, the algorithm’s global search capability is improved while simultaneously enhancing its local search ability. Equation (15) presents the computational formula for determining the contraction factor.

15 $$\text{\rm H}= \frac{2}{\left|2- \varnothing -\sqrt{{\varnothing }^{2}-4\varnothing }\right|}$$

where *ϕ* = c_1_ + c_2_ and *ϕ* > 4.

Also, to enhance the PSO’s convergence and global optimization performance, adaptive coefficients for inertia weight w and acceleration coefficients c_1_ and c_2_ are introduced^47^. These parameters dynamically change during the optimization process to balance exploration and exploitation according to Eq. (16).

16 $$w\left(t\right)= {w}_{max}- \frac{\left({w}_{max}- {w}_{min}\right)\times t}{{T}_{max}}$$

where w_max_ and w_min_ represent the maximum and smallest values of inertia weight, t denotes the current iteration, and T_max_ signifies the total number of iterations. The adaptive modification of inertia weight facilitates increased exploration during initial phases and encourages convergence in subsequent phases, thus averting premature convergence and enhancing global optimization efficacy.

**Figure:**
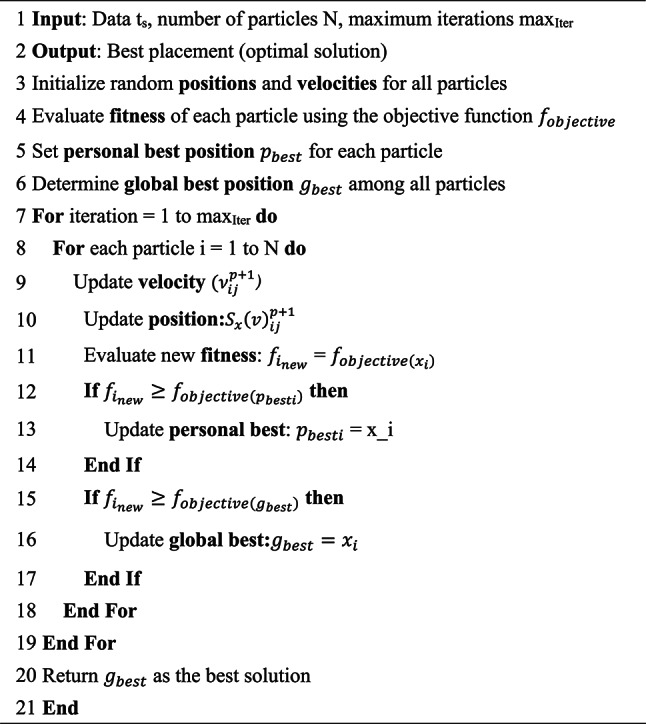
Algorithm 1

As indicated in Algorithm (1), Lines 1–3 present the algorithm’s inputs, outputs, and initiation phases. Line 4 evaluates the fitness of every particle. Lines five and six are meant to establish both local and global locations as well as particle fitness. Using ${\upnu }_{ij}^{p+1}$ and fitness function, each particle iteratively runs from 1 to the maximum number of iterations in lines 7–11 to compute location. d location. These processes change the velocity of every particle in line with its existing velocity, local ideal location, and global optimum position. The inertia of the particle, cognitive component (personal experience), and social component (collective experience) determine the new velocity. Lines 11–16 evaluate if the fitness of the new location exceeds the particle’s current local maximum fitness and update the local best position to the new one should the new position be better (line 13); should the new position be inferior, keep the local best position (line 15). Line 16 confirms whether the fitness of the new position exceeds the global best fitness; if so, the global best position is changed to the new position. On the other hand, should the new position be less than the present global best position, the former line 20 preserves it.

The next phase defines every particle in the population’s starting position and speed. Every iteration uses Eqs. (12) and (13) to change the velocity and position of every particle. Also, every particle evaluates the ${f}_{objective}$. Update both p_besti_ and g_best_.

After optimal data placement is determined by MPSO algorithm, the next critical step involves leveraging this strategically placed data to perform accurate tumor detection and classification directly at the fog nodes. Efficient placement of medical data to suitable fog nodes significantly reduces data processing latency, making real-time medical diagnostics feasible and improving patient outcomes in urgent clinical scenarios.

### Data processing phase

With the optimized distribution of data tasks to the appropriate fog nodes established, the framework proceeds seamlessly into the data processing stage. In this phase, the selected fog nodes utilize advanced image processing and deep learning techniques for reliable tumor detection and classification. The key steps involved in classifying a tumor at the fog node include:

- Preprocessing
- Feature extraction
- Data segmentation
- Classification

This sequential processing ensures the accuracy and timely availability of diagnostic outcomes, fully leveraging the benefits of strategically optimized data placement.

**Fig. 4:**
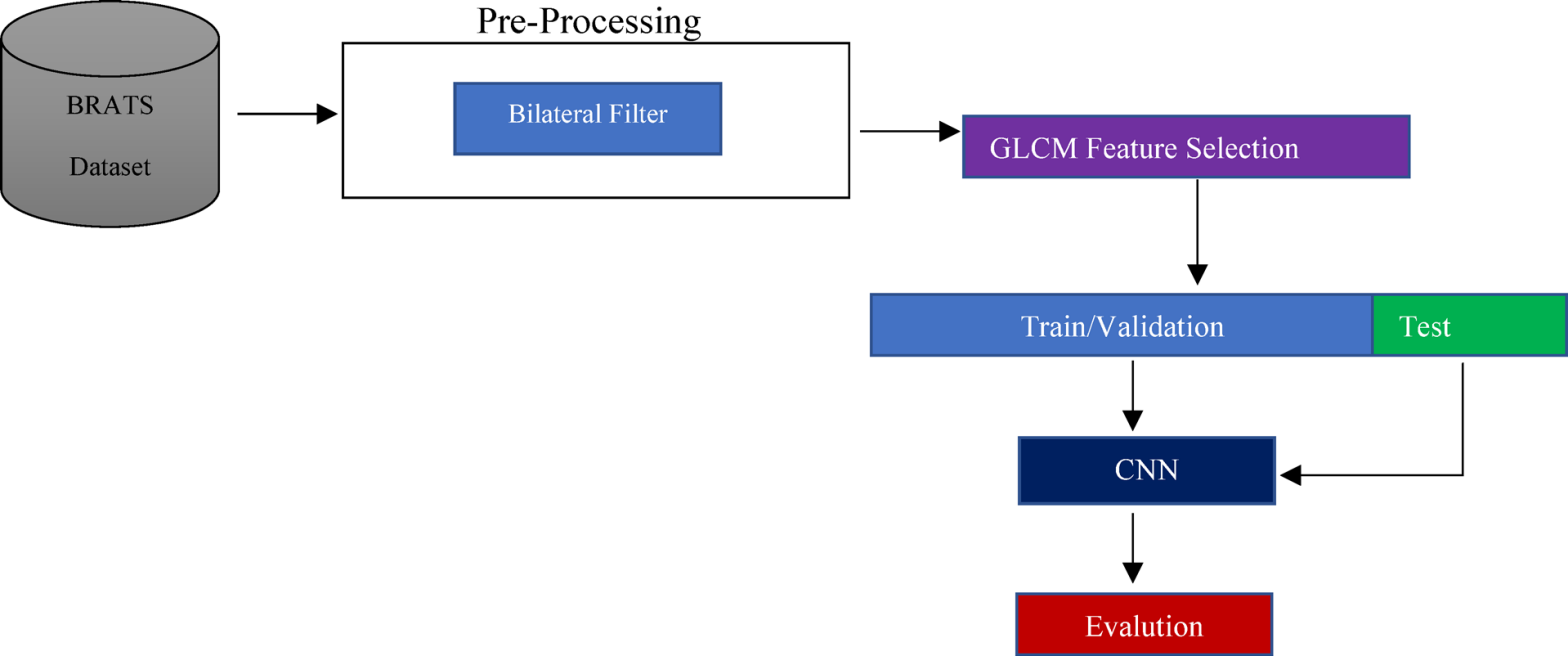
The Structure of the proposed method.

The method is structured into several units, as illustrated in Fig. 4. Initially, a Gaussian filter is employed during the preprocessing phase to enhance image quality and eliminate noise. Subsequently, the Gray Level Co-occurrence Matrix (GLCM) is utilized to extract texture information, a method commonly applied in healthcare processing and analysis systems due to its statistical nature. Moreover, significant features are selected and classified to predict the tumor with high accuracy. In the final phase, a deep CNN is implemented for classification.

### Pre-processing

In tumor detection applications, the quality and clarity of edges are very critical. Tumors typically manifest as distinct regions with varying intensities compared to brain tissue, and it is crucial to preserve the form and boundary integrity of these regions for feature extraction^48^. This method employs a bilateral filter to address these difficulties. The bilateral filter enhances it by eliminating high-frequency noise while preserving the fundamental edges of tumor formation and retaining the precise texture features for classification. Medical image processing, particularly in brain tumor identification, significantly benefits from bilateral filters that provide nonlinear, edge-preserving noise reduction capabilities. Conventional filtering methods uniformly smooth images, diminishing significant edges, but the bilateral filter employs a dual criterion to selectively apply smoothing to areas that are geographically and intensity similar^49^. The filter’s remarkable attribute is particularly beneficial in brain MRI analysis, as it aids in identifying and differentiating diagnostic tissue borders between normal brain tissue and malignancies.

For grayscale image I_f_, the bilateral filter calculates a weighted average of neighboring pixels to get a new filtered image If. The primary principle is to average not only adjacent pixels but also those with similar intensity values. Thus, it is expressed as Eq. (17).

17 $$I_{f} \left( i \right) = \frac{1}{{W_{i} }}\mathop \sum \limits_{{\left\{ {j \in {\Omega }} \right\}}} I\left( j \right) \cdot G_{{{\sigma s}}} \left( {\left| {i - j} \right|} \right) \cdot G_{{{\sigma r}}} \left( {\left| {I\left( i \right) - I\left( j \right)} \right|} \right)$$

where:

- i is the index (coordinates) of the center pixel being filtered.
- j is the index of a neighboring pixel within a window Ω around i.
- I(j) is the intensity of the neighboring pixel j.
- |i – j| is the Euclidean distance between pixels iii and j.
- ∣I(i) − I(j)∣ is the Absolute intensity difference between pixel i and j.
- Gσ_s_ ​​is the Spatial Gaussian kernel (weights pixels based on distance).
- Gσ_r_ is the Range Gaussian kernel (weights pixels based on similarity in intensity).

Also, W_t_ is the normalization term to ensure all weights sum to 1. It is formulated as Eq. (18).

18 $${W}_{i}= {\sum }_{\left\{j \in\Omega \right\}}{{G}_{{\upsigma }_{s}}}\left(|i - j|\right)\cdot {{G}_{{\upsigma }_{r}}}\left(\left|I\left(i\right)- I\left(j\right)\right|\right)$$

The key parameter in the bilateral filter is shown in Table 2.

**Table 2:** Key parameters in the bilateral filter.

Parameter	Symbol	Role	Effect	Range
Spatial sigma	σ_s_ ​	Controls size of the spatial neighborhood	More spatial smoothing; considers distant pixels	3–10
Range sigma	σ_r_ ​	Controls sensitivity to intensity differences	More tolerant to intensity changes; blurs edges	20–80 (depends on image contrast)
Filter window size	d	Diameter of the filter kernel/window	Larger area considered; slower but smoother	5–15 (must be odd)

### Feature extraction

The primary goal of feature selection is to discern traits that enhance outcomes and exclude those that produce errors. To enhance the model’s accuracy and computational effectiveness, characteristics exhibiting a significant association with the target attribute are selected. We utilize the two-dimensional (2D) GLCM for feature selection^50^. The occurrence frequency of event x with y is denoted by the (x,y) element of the two-dimensional histogram referred to as GLCM. The angle θ (0 degrees horizontal, 90 degrees vertical, 45 degrees along the positive diameter, and 135 degrees along the negative diameter) and the relative distance (d) between two pixels are utilized to ascertain the presence of a pixel with intensity x in relation to another pixel j at a defined distance (d) and direction (θ). Grayscale and y-axes are also utilized. The predominant method for texture feature extraction is the GLCM. This method calculates the GLCM, from which the statistical texture features of energy, contrast, homogeneity, and correlation are derived. The hierarchically converted image is subsequently enhanced. The pseudocode of the GLCM method is shown in Algorithm 2.

**Figure:**
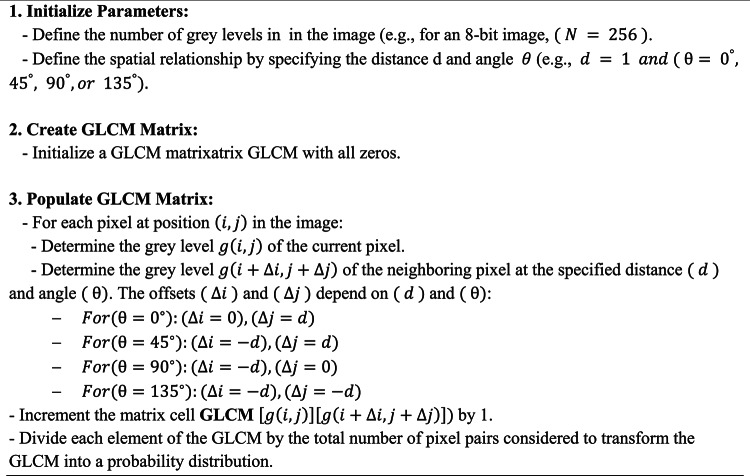
Algorithm 2

### Normalize GLCM matrix

**Fig. 5:**
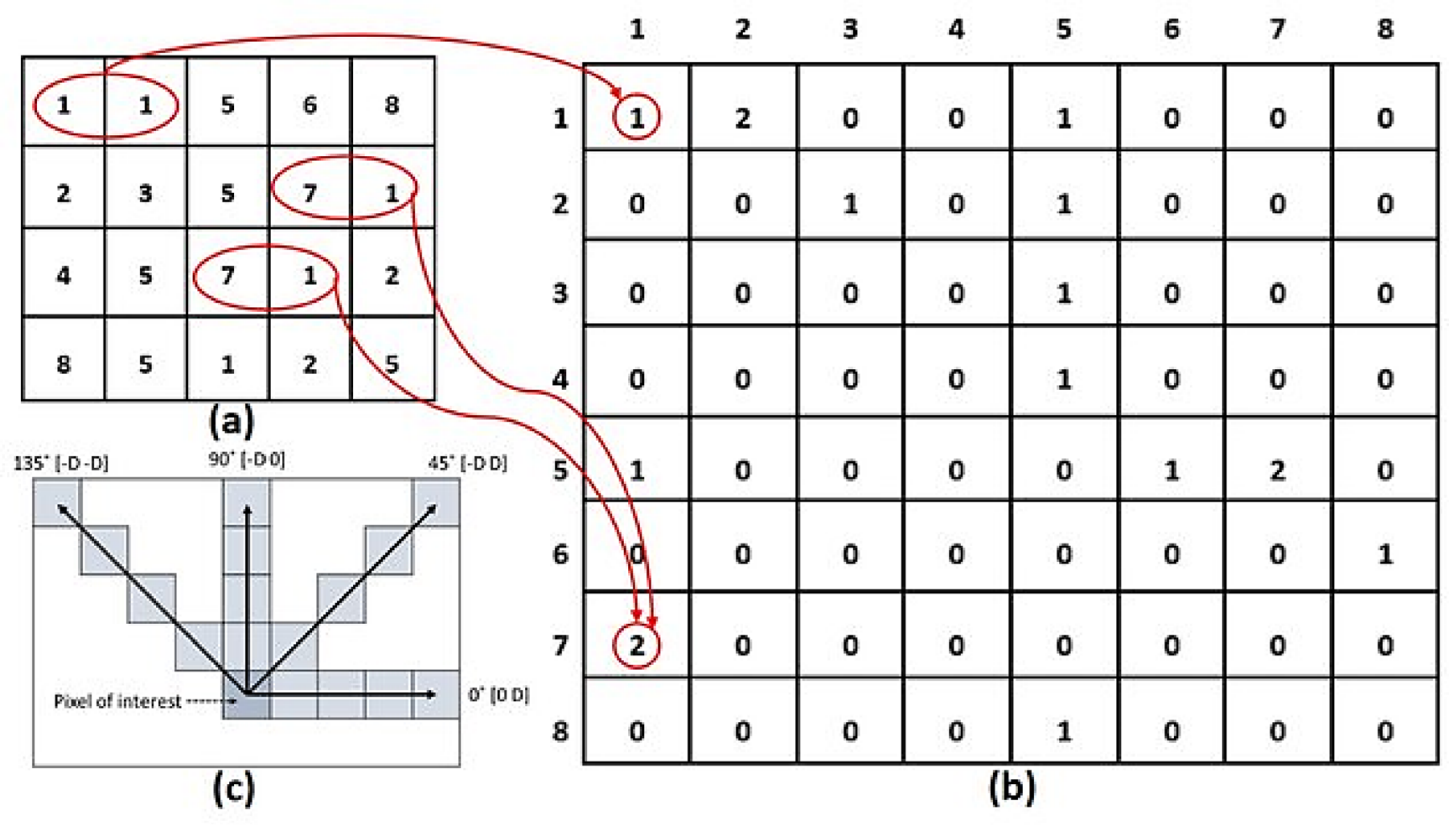
illustrates the procedure for processing the input image (**a**) into the GLCM image (**b**) using GLCM processing. The diagram (**c**) depicts the spatial arrangement of pixels in the array, specifically showing the row offsets and column offsets. In this context, D denotes the distance between each pixel of interest. The presence of red circles in the image indicates the frequency at which distinct combinations of gray levels occur. The arrows, on the other hand, represent the number of occurrences of these combinations in the GLCM picture.

Figure 5 depicts the methodology for computing the G matrix at a distance of 1 unit in the 0-degree direction. The GLCM matrix is constructed using a 4 × 5 matrix of the image depicted in this figure. This matrix comprises eight distinct color levels. The range (i,j) in the GLCM matrix denotes the frequency of pixels with color i in the image matrix, whereas the adjacent pixel color is j. The GLCM matrix is employed to obtain features or define specific regions of an image for testing and learning applications.

Three primary characteristics, namely "contrast, homogeneity, and energy," are utilized in accordance with Eqs. (19), (20) and (21) pertaining to the GLCM matrix. Equation (19) delineates the method for calculating contrast. The spatial frequency contrast of a picture is assessed. Contrast delineates the disparity between the maximum and minimum values inside a sequential array of pixels.

19 $$Contrast= \sum_{i=1}^{M}\sum_{j=1}^{N}(\left(i-j\right){ }^{2} \times p(i,j))$$

Also, image homogeneity is calculated through Eq. (20). In the pair of elements whose gray color has a small difference, its value will be large. Also, when all the elements in the image are the same, it will have the highest value.

20 $$Homogeneity= \sum_{i=1}^{M}\sum_{j=1}^{N}( \frac{p\left(i,j\right)}{1+\left|i-j\right|})$$

The energy computation is presented in Eq. (21). The technique computes the energy of the anomalies within the image textures, with a maximum attainable value of one. The maximum energy is observed when the gray-level distribution in the image is uniform or has a periodic pattern.

21 $$Energy= \sum_{i=1}^{M}\sum_{j=1}^{N}(p\left(i,j\right){ }^{2})$$

### Classification

**Fig. 6:**
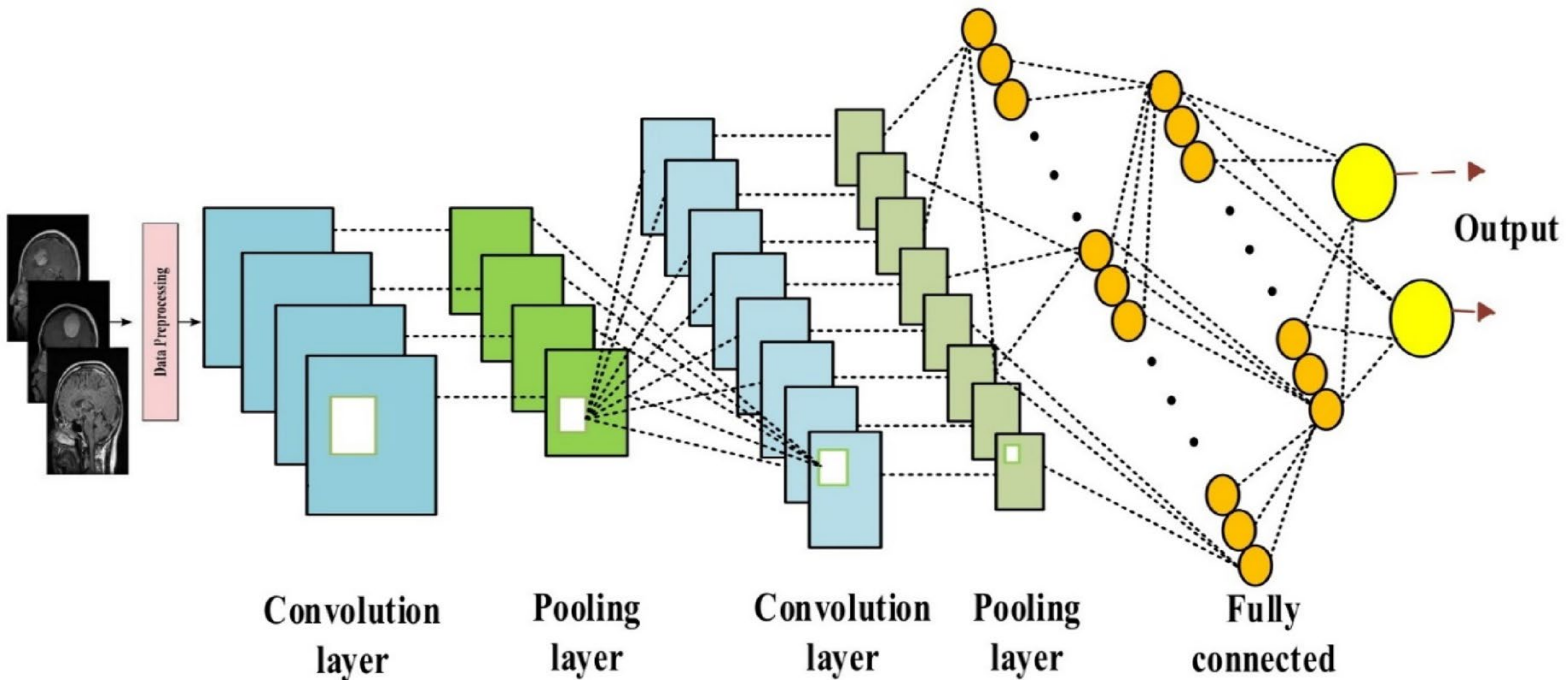
CNN algorithm structure.

This research employs a Convolutional Neural Network (CNN) with a sequential architecture, comprising an input layer, four convolutional layers, two pooling layers, and two fully connected layers, to classify brain tumors. The sequential CNN was chosen for its ability to effectively capture hierarchical spatial features from MRI images, which is critical for distinguishing complex tumor patterns in medical imaging^51^. Its layered architecture allows for progressive feature extraction, from low-level edges to high-level tumor-specific patterns, making it well-suited for the BraTS 2023 dataset’s multi-modal MRI scans. The sequential design also balances computational efficiency and model complexity, enabling deployment on resource-constrained fog nodes while maintaining high accuracy. Compared to alternative algorithms, such as Support Vector Machines (SVM), Random Forest, and other neural network architectures like DenseNet or PSP-Net, the sequential CNN offers superior performance for this task. SVM, while effective for smaller datasets, struggles with the high-dimensional, volumetric MRI data due to computational complexity and memory requirements, achieving lower accuracy^52^. Random Forest excels in tabular data but lacks the spatial feature extraction capabilities needed for image-based tumor detection, resulting in reduced accuracy^53^. DenseNet, although powerful for feature reuse, increases computational overhead, making it less efficient for real-time fog computing applications. PSP-Net, optimized for segmentation rather than classification, is less suitable for the final classification step, though it could complement the framework in segmentation tasks. As will be shown later, the sequential CNN achieves better accuracy and precision, outperforming these alternatives while maintaining computational efficiency suitable for fog nodes. Figure 6 illustrates its fundamental architecture, comprising an input layer, two pooling layers, two fully connected (FC) layers, and four convolutional layers. The convolution layer is the fundamental component of a CNN, tasked with processing data from a receiving cell.

The calculation for the output volume (Wo) size is presented in Eq. (22) and is defined as follows:

22 $${W}_{o}= \frac{{W}_{i}-S+2P}{P}+1$$

where the variables P, Wi, S, and M stand for the stride, input volume size, kernel size of the convolutional layer neurons, and amount of zero padding, respectively.

The function of the convolutional layer is to extract features from the data. It consists of many convolutional kernel layers, each linked to a weight and a bias coefficient. The weight coefficient, bi, and the input of convolutional layer I should correspond to X_j−1_, weight_i_, and b_i_, respectively, during the operation of the convolution kernel (i). A method to articulate the convolution process is equal to Eq. (23).

23 $$X_{j} = func\,(w_{j} + X_{{j - 1}} \otimes b_{j} )$$

where f(x) is the activation function, ⊗ denotes the convolution operation, and Xj is the output result of convolution kernel I.

The convolution kernel extracts details from the incoming data by periodic sweeping. Additionally, ReLU serves as the activation function for the convolutional layer. The ReLU activation function is more straightforward to derive than the sigmoid, tanh, and other activation functions. This facilitates the prevention of gradient vanishing and accelerates model training. The ReLU function can be expressed as Eq. (24).

24 $$Func_{R} eLU\left( {X_{j} } \right) = \left\{ {\begin{array}{*{20}l} {0 \left( {X_{j} \le 0} \right) } \hfill \\ {X_{j} \left( {X_{j} > 0} \right)} \hfill \\ \end{array} } \right.$$

The pooling layer eliminates redundant information from the CNN via down sampling, hence attaining invariance and decreasing CNN complexity^54^. Average pooling and maximal pooling are the two principal techniques for concluding pooling. In data pooling, average pooling utilizes the mean value of the designated region as the pooling outcome, whereas max pooling employs the maximum value of the area as the pooling result. The present study used the maximum pooling method, as it retains more significant information compared to average pooling. Max pooling is illustrated in Eq. (25):

25 $${Polling}_{r}=Maximum\left({p}_{r}^{1},{p}_{r}^{2},{p}_{r}^{3},\dots ,{p}_{r}^{n}\right)$$

where Maximum is the max pooling operation, p is the element (n) of the pooling area r, and ${Polling}_{r}$ is the output result of the pooling region j.

Subsequent to the convolutional layers, the ultimate representation of the extracted features is achieved by one or more fully connected layers. A fully connected layer resembles a convolutional layer; however, it is characterized by a comprehensive link to its preceding layer, in contrast to the sparse interactions typical of conventional neural networks. The last layer generates a one-dimensional vector, with its length corresponding to the total number of categorization classes. This layer is tasked with executing classification. The network’s output is employed to calculate its error rate, which is subsequently used to determine the network’s parameters and aid in its training. During this procedure, the network’s output is evaluated against the correct answer with an error function, and the resultant error rate is calculated. The procedure for determining experimental error is delineated by Eq. (26).

26 $$L\left(C,\widehat{C}\right)= \frac{1}{n}\sum_{i=1}^{n}l\left({C}_{i},f\left({X}_{i},w\right)\right)$$

The cost function L(C, $\widehat{C}$) measures the penalty associated with inaccurately forecasting $\widehat{C}$ in place of C. The post-propagation phase commences according to the calculated error rate in the following stage. In this phase, the gradient of each parameter is calculated via the chain rule, and all parameters are modified according to their influence on the error produced in the network, employing Eq. (27).

27 $${w}^{t+1}={w}^{t}-{\rho }_{t}\frac{\partial f}{\partial w}\left({w}^{t}\right)$$

**Fig. 7:**
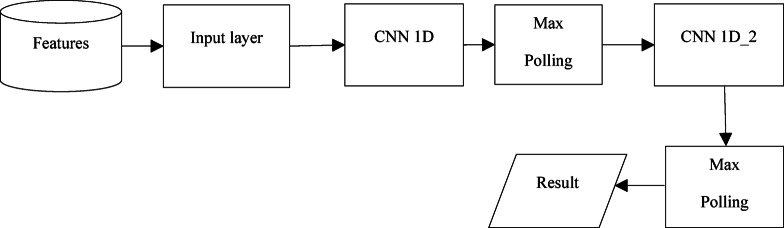
Flow diagram of proposed model.

Upon adjusting the parameters, the subsequent stage of feedforward begins. Upon the completion of a designated sequence of these stages, the network training process concludes. The fully connected layers function as the primary classifiers of the convolutional neural network. Their main responsibility is to reconfigure the characteristics of the pooling and convolutional layers from the hidden-layer domain to the sample-marker domain through weighting. To prevent overfitting, a dropout mechanism is implemented in the fully connected layer to randomly eliminate neurons. The model incorporates a sequential architecture with one-dimensional (1D) CNN techniques. Figure 7 illustrates the flow diagram of the proposed paradigm.

In the constructed sequential model, the input layer is positioned first. This layer delineates the dataset’s size. Figure 7 demonstrates the application of two CNN algorithms. The parameters of the CNNs were adjusted to achieve optimal results. Table 3 presents these parameters in a tabular format. The pooling layer was applied subsequent to the convolution process. The major goal of the pooling layer is to reduce the dimensionality of the retrieved feature matrix. Essential information is preserved in the pooling layer, thus reducing the processing strain^55^. Max pooling was employed during the first and second CNNs, while average pooling was utilized after the second CNN.

28 $${\text{Func}}\left( {\text{x}} \right) \, = {\text{ maximum}}\left( {0,{\text{x}}} \right)$$

**Table 3:** The CNN parameters.

	Filters num	Size of kernel	Activation
CNN D1	128	8	ReLu
CNN D1_2	64	4	ReLu

In both CNN algorithms, the activation function was the ReLu Eq. (28). Because it removes negative numbers and simplifies computation, the ReLu function was used.

## Dataset

For the development and validation of the models, we use BraTS 2023 dataset^56^. The BRATS dataset represents a standard benchmark that medical imaging specialists use to build and test their methods for brain tumor detection from MRI data. The MICCAI BRATS challenges introduced this dataset, which contains multi-institutional, multi-modality MRI scans of patients with glioma with high-grade (HGG) and low-grade (LGG). Researchers can analyze different tumor sub-regions, such as the enhancing tumor along with peritumoral edema and necrotic core, through T1 and T1ce and T2 and FLAIR MRI modalities in each scan. Neuroradiology experts segment patients’ brain tumors by hand to yield high-quality ground truth annotation data. The BraTS parameters are shown in Table 4.

**Table 4:** BraTS dataset parameters.

Parameter	Typical value/Description
Modalities	T1, T1ce, T2, FLAIR
Image size	240 × 240 × 155 voxels (after preprocessing)
Voxel spacing	1 mm^3^ (isotropic, after resampling)
Classes (Segmentation)	0 = Background, 1 = Necrotic core, 2 = Edema, 4 = Enhancing tumor
Combined regions	WT (Whole Tumor), TC (Tumor Core), ET (Enhancing Tumor)
Normalization	Z-score normalization per modality (zero mean, unit variance)
Input channels	4 (corresponding to the 4 modalities)
Label format	Single 3D volume per case with integer labels
Data format	NIfTI (.nii.gz)
Typical DL input shape	(Batch, Channels = 4, Depth = 155, Height = 240, Width = 240)
Common architectures	3D U-Net, V-Net, nnU-Net, DeepMedic
Loss functions	Dice loss, Cross-Entropy, Tversky loss, Combo loss
Evaluation metrics	Dice Similarity Coefficient (DSC), Hausdorff Distance, Sensitivity, Specificity
Augmentations	Rotation, flipping, scaling, elastic deformation, intensity shifts
Preprocessing	Skull stripping, co-registration, resampling, intensity normalization
Typical dataset split	80% training, 10% validation, 10% test (varies by experiment)

In the BraTS 2023 dataset used for model training, LGG includes both WHO grade 2 and grade 3 tumors as a unified class. This categorization reflects a widely adopted practice in neuro-oncology research, where grade 2 and 3 gliomas are often grouped due to their similar imaging characteristics and overlapping clinical management pathways. The proposed model, therefore, focuses on distinguishing major tumor categories that are clinically actionable, such as LGG versus HGG, rather than performing fine-grained grading. This aligns with practical diagnostic needs where early detection and broad-grade classification are critical for initiating timely treatment. However, we recognize that sub-classification of LGG into grades 2 and 3 could further support treatment planning. Future works of the model may explore integration with histopathological or genomic data to enable more detailed stratification without sacrificing the system’s real-time performance at the fog layer.

## Implementation

In this section, we give a detailed account of the simulation configuration and performance analysis of the proposed method, according to the results obtained from the performed experiments. The computed results illustrate the efficiency and applicability of the algorithm for different cases, which demonstrates the suitability of the algorithm in different parameters and conditions. We will also address the consequences of these results on further studies and applications. These results not only improve our understanding of the algorithm, but also potentially lead to novel applications.

### Experimental configurations

The research used a simulation-based approach to do trials with various parameter configurations. The proposed approach is rigorously evaluated using iFogSim2, a simulator built on the CloudSim platform^57^. This study seeks to assess the effectiveness of the proposed technique by allocating a mobile device to manage the data of a delay-sensitive application amidst an increasingly expanding user base. The aforementioned programs are organized as a collection of requests that are executed independently on virtual machines within the simulators. For a comprehensive understanding of the application model, including its components and interconnections, please refer to references^58,59^. The simulation parameters and their values for the model are shown in Table 5.

**Table 5:** Simulation setup.

Parameter	Description	Value/Range
Population size (n)	Number of particles in PSO	80
Iterations (maxIter)	Maximum iterations for convergence	50
VMs	Number of virtual machines	30–90
Data volume	Number of data tasks in simulation	150–800 (step size 150)
Dimension (dim)	Number of variables for optimization	3
PSO parameters (c1, c2)	Acceleration coefficients	1.0 each
Inertia weight (w_min_, w_max_)	Lower and upper bounds for inertia	0.4–0.9
Sigmoid threshold	Probability cutoff for binary PSO	0.5
VM placement metric	Resource-aware sorting criteria	Based on CPU, memory, bandwidth
Evaluation metric	Optimization objective	Weighted latency and energy cost

Tables 6 and Table 7 also list the characteristics of the fog nodes and the respective gateways, respectively.

**Table 6:** Fog devices characteristics.

CPU power	50,000	MIPS
Memory level	48,000	K.B
Bandwidth	12,000	bps
Energy usage when operating in active mode	1700	Joule
Energy usage when operating in idle mode	1100	MIPS
Number of data	Variable

**Table 7:** Gateways characteristics.

CPU power	2700	MIPS
Memory level	4200	K.B
Bandwidth	13,000	bps
Energy usage when operating in active mode	120	Joule
Energy usage when operating in idle mode	2700	MIPS

#### Comparison methods

The selected methods for comparison are Ant Colony Optimization (ACO), Shortest Job First (SJF), Round Robin (R.R), Genetic and Simulated Annealing (GASA), Harris Hawks Optimization (HHO), Enhanced African Vultures Optimization algorithm (EAVOA), Deep Deterministic Policy Gradient (DDPG), Genetic Algorithm (GA)^60–62^, and the Proposed Method (HETS-IP). Metaheuristic parameter values are presented in Table 8.

**Table 8:** Metaheuristic parameters.

Algorithm	Parameters value
PSO	C_1_, C_2_: 1 w_min_, w_max_: 0.4, 0.9 Population (n): 50 Iteration: 30 Dimension (dim): 3
ACO	Num of Ants: 50 a: 3 b: 2 r: 0.02
Genetic	Population size: 50 Selection rate: 0.7 Crossover rate: 0.7 Mutation rate: 0.3 Generation: 30
GASA	Annealing function: Fast annealing Temperature update function: Linear Reannealing interval: 600–1500 Initial temperature: 25–85
HHO	Num of agents: 50 Max Iteration: 30
EAVOA	Population size: 50 Iteration: 30 P1: 0.6 P2, P3: 0.5 L1: 0.4, L2: 0.6

### Test 1

**Fig. 8:**
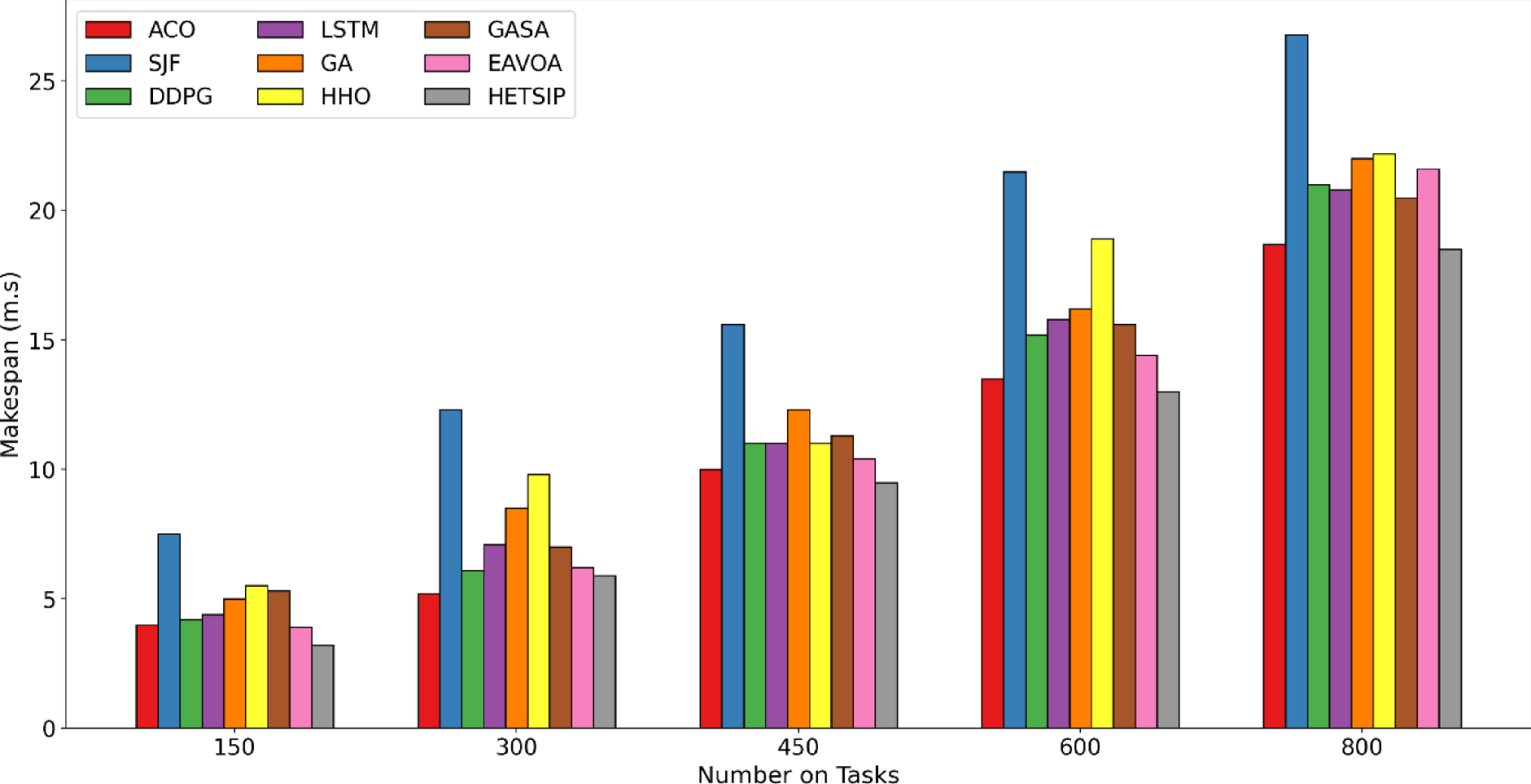
**C**omparison of makespan in test 1.

**Fig. 9:**
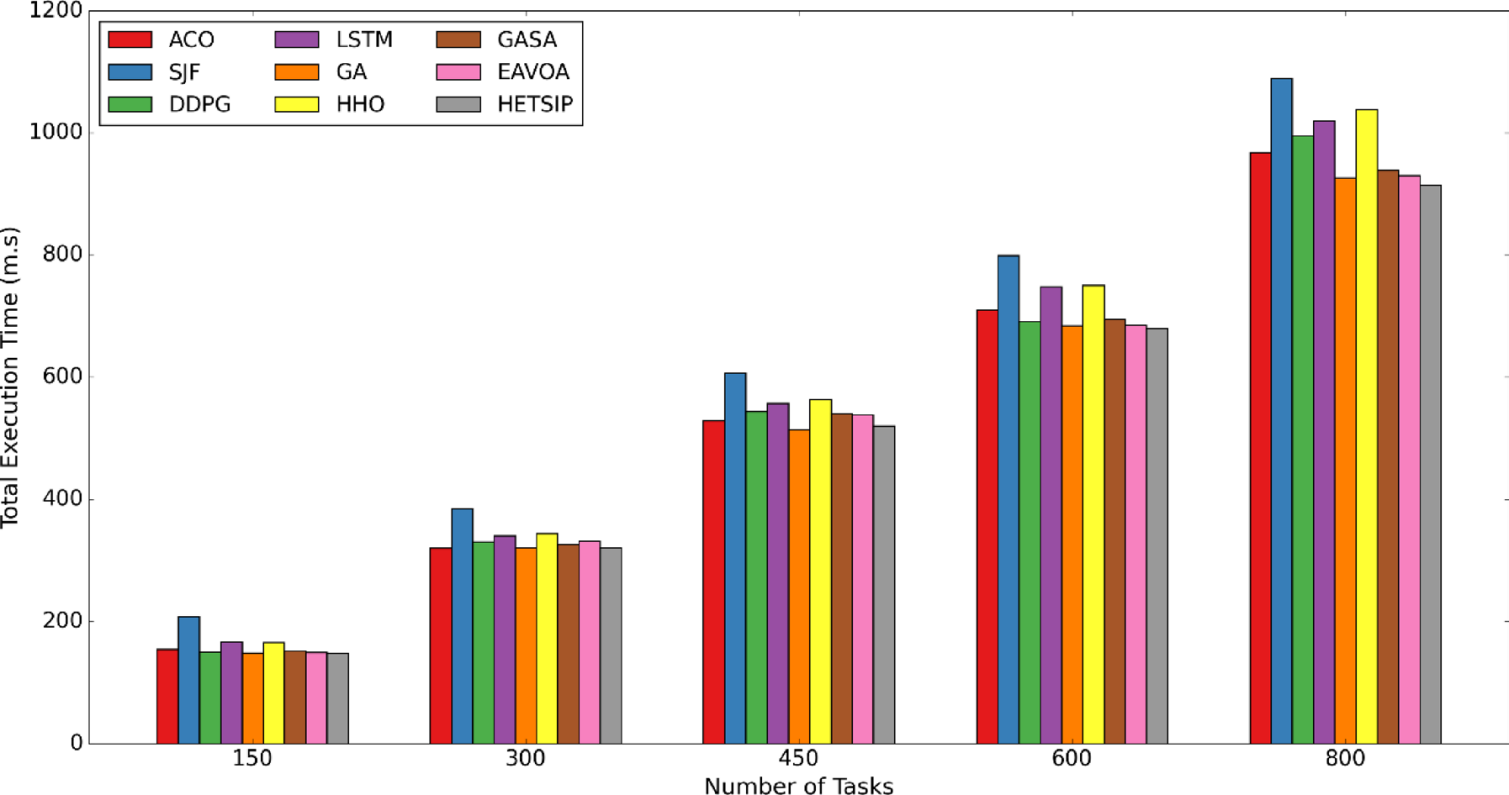
**C**omparison of execution time in test 1.

**Fig. 10:**
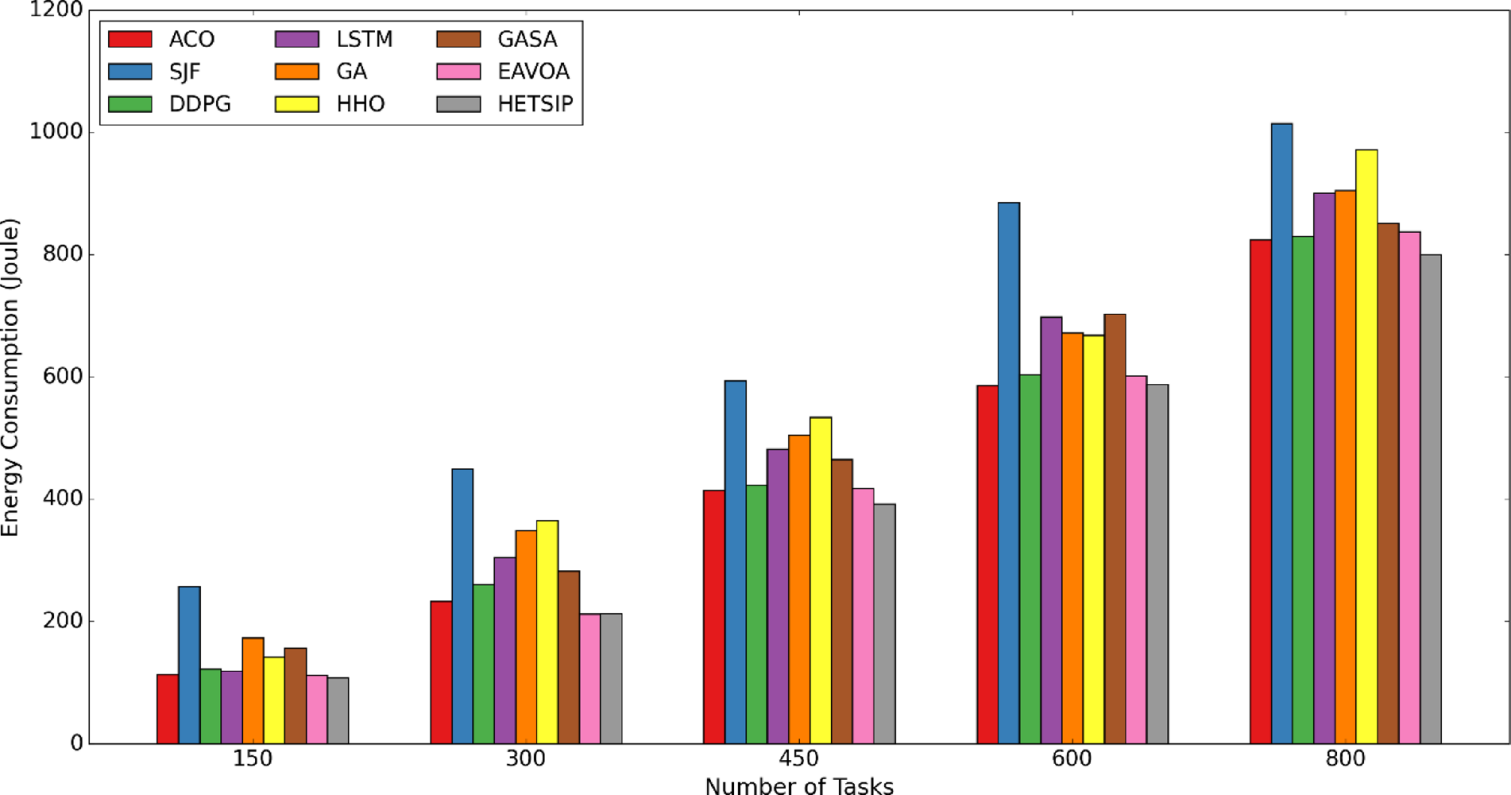
**C**omparison of energy consumption in test 1.

In Test 1, the data quantity is consistently adjusted within a range of [150–800], in increments of 150. A consistent number of 60 virtual machines is upheld during the experiment. Figures 8, 9 and 10 present bar charts that depict the comparative performance of algorithms regarding cumulative execution time, makespan, degree of imbalance, and energy consumption. Reducing makespan improves throughput and service quality by allowing the system to process more data in resource-limited fog conditions. Minimizing energy consumption mitigates node overloads, hence assuring cost-efficiency, system dependability, and sustainability. Reducing execution time is essential for satisfying low-latency demands in time-sensitive applications. Efficient load balancing enhances scalability, fault tolerance, and resource consumption, guaranteeing optimal data processing throughout the system.

The examination of makespan performance, depicted in Fig. 8, reveals a distinct trend: the makespan escalates with the increasing volume of data across all algorithms. This is anticipated, as handling increased data typically results in extended processing durations. Nonetheless, HETS-IP distinguishes itself by continuously attaining the minimal makespan across all data sets. The dual-phase strategy, which optimizes virtual machine deployment and subsequently data placement using an advanced PSO algorithm, guarantees more efficient data management, leading to reduced overall completion times. Conversely, the SJF algorithm consistently demonstrates the largest makespan values in all cases. This inefficiency arises from SJF’s emphasis on prioritizing shorter data while neglecting the total system load and the potential delays affecting longer data. It is far more efficient than the two recent methods, specifically the DDPG and EAVOA.

HETS-IP attains a reduced makespan relative to DDPG and EAVOA by optimizing data and VM placement, hence directly diminishing execution time. DDPG prioritizes dynamic load balancing, EAVOA underscores overall optimization, whereas HETS-IP utilizes an advanced PSO algorithm with direct binary encoding to accurately allocate data to VMs and reduce idle times. Furthermore, its virtual machine placement technique, which classifies actual computers according to resource availability, guarantees optimal allocation and prevents bottlenecks. These customized capabilities allow HETS-IP to manage data more effectively, leading to a continually reduced makespan. Also, Compared to traditional evolutionary methods such as ACO, GA, and GASA, HETS-IP exhibits superior performance due to several key enhancements. First, while GA and ACO rely on iterative random search and pheromone trails, respectively, they tend to converge slowly and can become trapped in local optima, especially in high-dimensional or discrete task placement scenarios. GASA, though improved with simulated annealing for better convergence control, still suffers from high computational overhead due to its hybrid nature. Essentially, the shorter makespan that HETS-IP achieves allows for faster processing and analysis of vital medical data, like brain scans or sensor readings, which facilitates quicker diagnosis and shorter decision cycles in healthcare systems. This efficiency can result in better patient outcomes, faster clinician response times, and increased system scalability as data loads rise in high-throughput and emergency settings like remote diagnosis units or intensive care unit monitoring. Additionally, shorter processing times result in lower energy requirements, which is essential for Internet of Things-based medical devices that operate in environments with limited power, like mobile diagnostic units or rural clinics.

As a result, the makespan increases significantly, particularly as the number of data grows. This comparison underscores HETS-IP’s superior ability to handle varying workloads effectively, minimizing the makespan and enhancing overall system performance.

The comparison experiments illustrated in Fig. 9 demonstrate that HETS-IP and GA are the most efficient algorithms for reducing total execution time across different data volumes in fog computing environments. HETS-IP consistently attains the minimal execution times at 150, 300, 600, and 800 data points, demonstrating its durability and efficiency. GA excels at 450 data and EAVOA at 150; however, this advantage is not sustained across all scenarios, suggesting that while competitive, HETS-IP exhibits superior consistency. The findings underscore HETS-IP’s enhanced scalability and adaptability, efficiently handling bigger workloads and maximizing resources used more consistently than GA, ACO and EAVOA. In contrast, the SJF algorithm consistently exhibits the longest overall execution durations across all data volumes, indicating its inefficiency in managing bigger workloads due to its emphasis on shorter tasks without regard for system load. Likewise, the Round Robin and HHO algorithms are positioned slightly above SJF, exhibiting comparatively prolonged execution times owing to their rudimentary data allocation methodologies. The findings indicate that sophisticated algorithms such as HETS-IP, GASA, GA, and EAVOA are favored for enhancing performance in fog computing, whereas simpler methods like SJF and R.R. may be less successful for intricate, large-scale data.

Also, Reduced execution time directly supports the provision of real-time diagnostic services in healthcare systems. Rapid data analysis is essential for medical situations like tumor detection, patient monitoring, and emergency triage in order to support decisions that could save lives. HETS-IP enables a quicker transition from sensor input to diagnostic insight by guaranteeing low-latency execution across large, heterogeneous workloads, enhancing the clinical responsiveness of intelligent healthcare systems.

Figure 10 illustrates an analysis of energy consumption among several algorithms, indicating that energy usage escalates with the rise in data volume. Among the assessed algorithms, HETS-IP consistently demonstrates the lowest consumption of energy across all data volumes, underscoring its resource management efficiency. The minimal energy use is a direct consequence of HETS-IP’s optimized data and VM placement procedures, which reduce superfluous energy expenditure. Conversely, the SJF, HHO, and LSTM algorithms consistently exhibit the highest energy consumption in all cases. This pattern corresponds with their performance in other metrics, where they similarly show inefficiencies. The elevated energy consumption of SJF and LSTM underscores their suboptimal management of data and resources, resulting in heightened operating expenses and diminished overall efficiency. HETS-IP is the most efficient in terms of energy algorithm, rendering it very beneficial in fog computing scenarios where conserving energy is paramount. The persistent underachievement of SJF, HHO, and LSTM across several measures, including energy usage, highlights their deficiencies in resource optimization, hence reinforcing HETS-IP as the preeminent option for effective data placement and managing resources. Specifically, as illustrated in Fig. 10, HETS-IP exhibits enhanced energy efficiency relative to DDPG and EAVOA by integrating efficient data placement with an energy-conscious VM placement strategy. HETS-IP classifies real machines according to resource availability, ensuring data is assigned to the most appropriate VMs, hence minimizing resource waste and avoiding the unnecessary deployment of additional machines. Conversely, DDPG and EAVOA exhibit an absence of comprehensive data allocation strategies, resulting in increased energy consumption. Consequently, HETS-IP constantly attains reduced energy consumption while sustaining superior performance. HETS-IP also outperforms conventional metaheuristics like GA, GASA, and ACO. These approaches either need more iterations to converge or do not explicitly incorporate energy constraints into their objective functions, which raises the overall energy consumption. On the other hand, HETS-IP prioritizes low-energy configurations in its solution space and enables faster convergence through the use of adaptive inertia control and direct binary encoding within its PSO core.

These energy savings have important ramifications for healthcare applications. Numerous medical Internet of Things devices, like wearable monitors or portable diagnostic sensors, function in energy-constrained settings where it is impractical to replace or recharge batteries frequently, particularly in disaster areas, mobile health units, and rural clinics. HETS-IP contributes to increased system reliability and operational sustainability by extending device uptime and reducing infrastructure strain through the reduction of energy expenditure. Additionally, it lowers long-term energy expenses for medical facilities that implement fog-based systems on a large scale.

Tables 9, 10 and 11 display the numerical outcomes corresponding to each illustration.

**Table 9:** Numeral results of makespan in test1.

Data	Algorithms								
	ACO	SJF	DDPG	GA	LSTM	HHO	GASA	EAVOA	HETS-IP
150	4.0	7.5	4.2	5.0	4.4	5.5	3.2	3.9	5.3
300	5.2	12.3	6.1	8.5	7.1	9.8	5.9	6.2	7.0
450	10.0	15.6	11.0	12.3	11.0	11.0	9.5	10.4	11.3
600	13.5	21.5	15.2	16.2	15.8	18.9	13.0	14.4	15.6
800	18.7	26.8	21.0	22.0	20.8	22.2	18.5	21.6	20.5

**Table 10:** Numeral results of execution time in test1.

Data	Algorithms								
	ACO	SJF	DDPG	LSTM	GA	HHO	GASA	EAVOA	HETS-IP
150	148.0	148.9	151.5	165.8	148.5	166.5	150.2	208.5	153.9
300	320.8	332.0	326.0	344.0	320.8	340.7	330.8	384.7	320.5
450	520.5	538.5	540.8	563.0	514.8	556.5	544.1	606.0	529.1
600	679.2	684.7	694.5	750.2	683.9	748.0	690.5	798.9	709.5
800	914.0	930.0	938.5	1038.1	926.2	1019.5	995.0	1089.2	967.3

**Table 11:** Numeral results of energy consumption in test1.

Data	Algorithms								
	ACO	SJF	DDPG	LSTM	GA	HHO	GASA	EAVOA	HETS-IP
150	108	112	157	142	173	119	122	257	113
300	213	212	283	365	349	305	261	450	233
450	392	418	465	534	505	482	423	594	415
600	588	602	703	668	672	698	604	885	586
800	800	838	851	972	905	901	830	1015	824

### Test 2

This section describes standard evaluation metrics for deep learning models. Confusion matrix can be used to describe the performance of the classifier in a 2D tabular form (stored in the form of a table). It comprises the TP, FN, FP and TN values. These are then the subsequent foundation of a number of metrics. These metrics based on the values in the confusion matrix provide a measurement of model performance with regard to accuracy, precision, recall and F1-score. These metrics, derived from the values in the confusion matrix, help quantify the model’s performance. By analyzing these metrics, practitioners can gain insights into how well their deep learning model is performing and where improvements may be needed.

29 $$Accuracy=\frac{TP+TN}{TP+TN+FP+FN}$$

30 $${\text{Precision}} = \frac{TP}{{{\text{TP}} + {\text{FP}}}}$$

31 $${\text{Recall}} = \frac{TP}{{TP + FN}}$$

32 $$F1-Score=\frac{2*Precision*Recall}{Precision+Recall}$$

33 $$\text{TNR }=\frac{TN}{\text{FP}+\text{TN}}$$

34 $$\text{MCC }=\frac{\left(TP\times TN\right)-\left(FP\times FN\right)}{\sqrt{\left(TP+FP\right)\left(TP+FN\right)\left(TN+FP\right)\left(TN+FN\right)}}$$

The algorithms for image pre-processing, feature extraction, and CNN based classification have been implemented using MATLAB R2022 environment. Tables 12 and 13 display the outcomes of CNN classification for the 3 distinct categories of brain tumor based on BRATS dataset.

**Table 12:** Performance of CNN classification based on level 1 GLCM.

Sets	Accuracy (%)	Precision (%)	Recall (%)	F1-Score (%)	MCC (%)
Low-grade glioma (LGG)	96.0	97.4	95.1	96.1	91.1
Glioblastoma (HGG)	96.1	94.4	97.6	96.2	91.3
No tumor	87.0	96.8	78.1	82.5	71.5

**Table 13:** Performance of CNN classification based on level 2 GLCM.

Sets	Accuracy (%)	Precision (%)	Recall (%)	F1-score (%)	MCC (%)
Low-grade glioma (LGG)	93.5	95.2	92.4	93.6	84.8
Glioblastoma (HGG)	93.7	93.5	94.1	92.8	84.4
No tumor	85.3	97.4	78.5	82.4	71.5

We have employed a method to differentiate various categories based on their grades: Low-Grade Glioma (Grade 1), Glioblastoma (Grade 2), and No tumor (grade 0). LGG is a combined category of WHO grade 2 and grade 3 gliomas and this type of LGG tumors is less aggressive, malignant brain tumors, and High-Grade Glioma (HGG) refers only to WHO grade 4 gliomas (Glioblastoma), which are well-known Grade 2 gliomas grow more slowly as compared to Grade 3 or anaplastic gliomas which are more aggressive but not as much as Grade 4^63^. As the imaging features of Grades 2 and 3 are usually very similar BraTS combines them to LGG, thus making the classification tasks to LGG vs HGG without clearly discriminating Grade 2 from Grade 3. We have extracted six texture features (dissimilarity, contrast, entropy, energy, homogeneity, and correlation) from sampling images. A thorough analysis of this research reveals that image texture encompasses significant diagnostic information essential for the processing and differentiation of benign and malignant tumors in medical imaging. However, the proposed features are insufficient for distinguishing between benign and malignant tumors; it is imperative to identify and differentiate them using more reliable indicators.

**Fig. 11:**
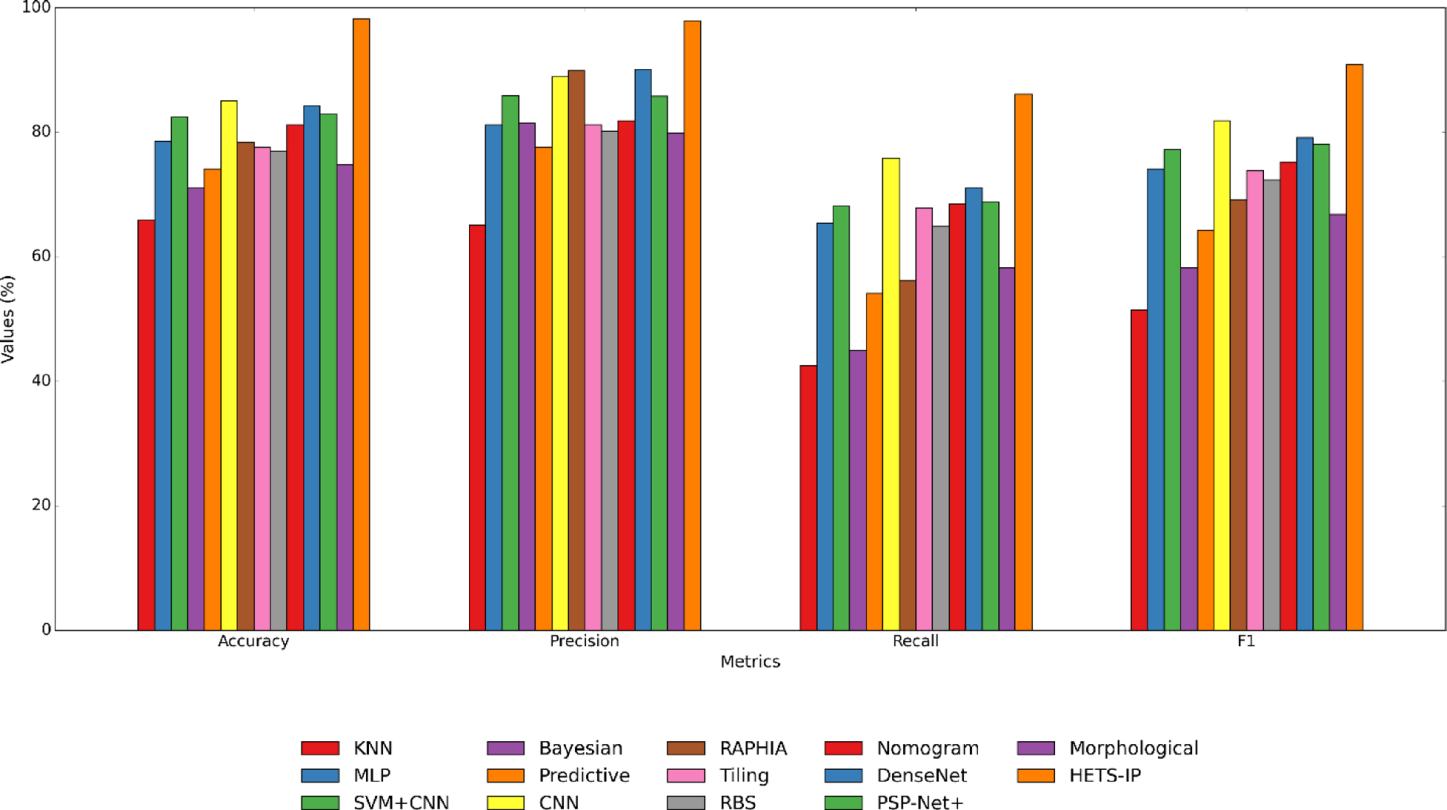
Comparison of the methods.

Furthermore, Fig. 11 and Table 14 are dedicated to comparing HETS-IP with alternative approaches regarding Accuracy, Precision, Recall, F1 Score, and AUC. The stated outcomes encompass the average values compiled from various executions of each program. The proposed approach has been compared with analogous methods, the majority of which pertain to tumor detection. Accuracy quantifies the model’s overall correctness by computing the ratio of properly predicted occurrences to the total number of instances. Precision is the proportion of accurately predicted positive observations to the total expected positives, indicating the model’s capacity to minimize false positives. Recall is the ratio of accurately predicted positive observations to all actual positives, reflecting the model’s capacity to identify all pertinent cases (true positives). The F1 score represents the harmonic means of precision and recall, offering a balanced metric that accounts for both false positives and false negatives.

**Table 14:** Comparison of the HETS-IP with other methods.

Algorithm	Accuracy	Precision	Recall	F1-score
KNN	65.66	66.25	41.25	51.30
MLP	78.33	80.24	66.51	73.18
SVM + CNN	81.33	86.76	67.92	76.16
Bayesian	71.00	80.37	45.96	57.50
Predictive	73.00	78.66	53.11	63.33
CNN	85.00	89.82	76.81	82.80
RAPHIA	77.33	89.74	55.33	68.05
Tiling	78.66	80.26	68.66	74.00
RBS	77.00	79.27	65.96	71.38
Nomogram	80.33	82.95	67.38	74.34
DenseNet	83.33	90.00	70.10	78.20
PSP-Net +	83.00	86.61	69.69	77.21
Morphological	75.66	80.78	57.55	67.50
HETS-IP	97.33	98.00	85.13	91.05

The results reported in Table 5 demonstrate that the HETS-IP method surpasses its counterparts, which were also examined in this experiment. This better performance is related to the effectiveness of the feature extraction techniques applied, particularly the GLCM method. These strategies were essential in obtaining important textural information from tumor tissue images, considerably boosting the classifier’s capacity to reliably identify between histological grades of tumor. The influence of these advanced feature extraction approaches on the classifier’s success is both clear and large, as indicated by the remarkable accuracy and precision metrics achieved.

### Statistical analysis

To evaluate the significance of the reported enhancements, the results obtained from several iterations of each method were analyzed statistically using IBM SPSS V.26. Additionally, in conjunction with the typical calculation of descriptive statistics (mean ± SD), MANOVA and Tukey’s test were performed for each evaluation metric to ascertain any significant differences in the comparisons conducted. The selected significance level was 0.05. The MANOVA test aims to determine whether there is a significant difference among the outcomes. Tukey’s HSD test facilitates the comparison of each pair of means, allowing us to ascertain whether pairs have a significant difference. The results obtained from the MANOVA analysis are displayed in Table 15.

**Table 15:** Results of MANOVA test.

Measure	*f*-ratio	*p*-value
Accuracy	38.0528	< 0.00001
Precision	30.0156	< 0.00001
Recall	441.929	< 0.00001
F1	87.0745	< 0.00001
AUC	160.9321	< 0.00001

The MANOVA test results, presented in Table 15, indicate statistically significant differences among the means of the analyzed algorithms across all metrics: Accuracy, Precision, Recall, F1, and AUC. To conduct a pairwise analysis for further research, it is necessary to run the Tukey’s HSD test. The importance of the disparity between each algorithm pair presented in Table 16 was analyzed utilizing the HSD test. For this part of the statistical analysis, we concentrate exclusively on assessing the difference between the strategy that has yielded the most favorable results and its alternative methods. Table 8 presents the outcomes of the HSD test for three pairs, encompassing the proposed method and its nearest rivals. The table emphasizes the principal metric Q, commonly referred to as the studentized range statistic. Q is determined by using the pair of mean values under examination.

**Table 16:** The results of Tukey’s HSD test.

Pairwise comparisons	Q_.05_ = 4.242 Q_.01_ = 5.302				
(Method1 : Method2)	Accuracy difference	Precision difference	Recall difference	F1 difference	AUC difference
HETS-IP: DenseNet	Q = 16.20 (*p* = 0.00000)	Q = 7.75 (*p* = 0.00005)	Q = 41.36 (*p* = 0.00000)	Q = 20.20 (*p* = 0.00000)	Q = 26.93 (*p* = 0.00000)
HETS-IP: PSP-Net +	Q = 15.20 (*p* = 0.00000)	Q = 2.55 (*p* = 0.37817)*	Q = 45.55 (*p* = 0.00000)	Q = 18.07 (*p* = 0.00000)	Q = 30. 80 (*p* = 0.00000)
HETS-IP: CNN	Q = 11.27 (*p* = 0.00000)	Q = 0.58 (*p* = 0.99626)*	Q = 1.86 (*p* = 0.00000)	Q = 0.89 (*p* = 0.98175)*	Q = 11.10 (*p* = 0.00000)

*Non-significant difference.

The results of Tukey’s HSD test reveal a significant difference between the superior outcomes achieved by HETS-IP and those of its equivalents in most instances. However, there were a few instances where the difference was not deemed substantial. These exceptions arose for the PSP-Net + and CNN algorithms. The Tukey’s HSD test indicated that the enhancement in precision attained by the suggested method relative to the PSP-Net + method is not statistically significant. A comparable scenario is noted regarding the discrepancies between the outcomes of the HETS-IP and CNN algorithms, specifically with Precision and F1, which were statistically insignificant.

## Conclusions

The convergence of IoT and fog computing not only transformed the smart healthcare but also enabled real-time and low-latency diagnosis which are very critical in the case of time critical medical conditions like detection of brain tumor. This study suggested HETS-IP, a hybrid evolutionary framework improved with a modified PSO algorithm and direct binary encoding, to address the urgent issues of data placement, computational efficiency, and responsiveness in healthcare systems. The technique dramatically lowers latency, energy consumption, and processing time by strategically placing medical IoT data at the best fog nodes. According to simulation results, HETS-IP performs better than conventional algorithms in terms of makespan, execution time, and energy efficiency. These benefits are essential in real-world clinical settings where prompt and accurate processing of sensor and imaging data is necessary for ongoing monitoring, early diagnosis, and prompt medical intervention.

The implications of this approach go far beyond the detection of brain tumors and encompass a broad range of fog-based healthcare services. For example, it supports emergency response systems, remote chronic disease management, wearable health monitoring for high-risk or elderly patients, and real-time ECG or EEG analysis. In rural or resource-constrained environments, HETS-IP helps create scalable, resilient, and energy-conscious medical infrastructures by decreasing reliance on cloud servers and increasing local processing efficiency. Furthermore, the framework’s flexibility enables it to manage the high-frequency, context-sensitive, and heterogeneous data that characterizes contemporary healthcare IoT environments. Because of this, HETS-IP is positioned to play a key role in the creation of autonomous, decentralized healthcare ecosystems in the future. These ecosystems will be able to support AI-driven clinical decision support, anomaly detection, and predictive analytics. Although the suggested model performs well, there are some limitations that call for more research. First, the system has not yet been implemented or tested in real-world clinical settings, where hardware malfunctions and environmental fluctuations could affect performance. Instead, it is currently validated in a simulated environment. Second, the tumor detection framework may not be as generalizable to other imaging modalities or institutions because it was trained and validated on the BraTS dataset. Third, the current model ignores data security and privacy issues, which are crucial in clinical settings where sensitive patient data is handled. Future research will concentrate on incorporating mobility-aware and adaptive resource allocation strategies, extending the system to accommodate cross-institutional and multimodal datasets, integrating privacy-preserving measures like secure data access protocols and encryption, and investigating hybrid deep learning models for more comprehensive, context-aware health analytics in order to address these issues. The practicality, robustness, and clinical relevance of fog-based healthcare systems driven by HETS-IP are intended to be further improved by these developments. Also, future iterations of the proposed framework may incorporate explainable AI (XAI) techniques to highlight tumor-relevant regions within the MRI images. Methods such as Grad-CAM, saliency maps, or attention-based visualization can help radiologists and clinicians better understand the rationale behind the model’s predictions. Integrating these tools into the fog-based processing pipeline would not only improve transparency but also assist in validating segmentation boundaries and localizing tumor sub-regions. This aligns with the increasing demand for interpretable AI solutions in healthcare and complements the system’s diagnostic utility in real-time environments.

## Data Availability

The datasets used and/or analyzed during the current study available from the corresponding author on reasonable request. For validation of the models, we used BraTS 2023 dataset. The dataset is publicly available on platforms like Kaggle and The Cancer Imaging Archive.
